# Mapping the global potential distributions of two arboviral vectors *Aedes aegypti* and *Ae*. *albopictus* under changing climate

**DOI:** 10.1371/journal.pone.0210122

**Published:** 2018-12-31

**Authors:** Mahmoud Kamal, Mohamed A. Kenawy, Magda Hassan Rady, Amany Soliman Khaled, Abdallah M. Samy

**Affiliations:** 1 Entomology Department, Faculty of Science, Ain Shams University, Abbassia, Cairo, Egypt; 2 Research and Training Center on Vectors of Diseases, Faculty of Science, Ain Shams University, Abbassia, Cairo, Egypt; Instituto de Pesquisas de Rene Rachou, BRAZIL

## Abstract

**Background:**

*Aedes aegypti* and *Ae*. *albopictus* are the primary vectors that transmit several arboviral diseases, including dengue, chikungunya, and Zika. The world is presently experiencing a series of outbreaks of these diseases, so, we still require to better understand the current distributions and possible future shifts of their vectors for successful surveillance and control programs. Few studies assessed the influences of climate change on the spatial distributional patterns and abundance of these important vectors, particularly using the most recent climatic scenarios. Here, we updated the current potential distributions of both vectors and assessed their distributional changes under future climate conditions.

**Methods:**

We used ecological niche modeling approach to estimate the potential distributions of *Ae*. *aegypti* and *Ae*. *albopictus* under present-day and future climate conditions. This approach fits ecological niche model from occurrence records of each species and environmental variables. For each species, future projections were based on climatic data from 9 general circulation models (GCMs) for each representative concentration pathway (RCP) in each time period, with a total of 72 combinations in four RCPs in 2050 and 2070. All ENMs were tested using the partial receiver operating characteristic (*p*ROC) and a set of 2,048 and 2,003 additional independent records for *Ae*. *aegypti* and *Ae*. *albopictus*, respectively. Finally, we used background similarity test to assess the similarity between the ENMs of *Ae*. *aegypti* and *Ae*. *albopictus*.

**Results:**

The predicted potential distribution of *Ae*. *aegypti* and *Ae*. *albopictus* coincided with the current and historical known distributions of both species. *Aedes aegypti* showed a markedly broader distributional potential across tropical and subtropical regions than *Ae*. *albopictus*. Interestingly, *Ae*. *albopictus* was markedly broader in distributional potential across temperate Europe and the United States. All ecological niche models (ENMs) were statistically robust (P < 0.001). ENMs successfully anticipated 98% (1,999/2,048) and 99% (1,985/2,003) of additional independent records for both *Ae*. *aegypti* and *Ae*. *albopictus*, respectively (P < 0.001). ENMs based on future conditions showed similarity between the overall distributional patterns of future-day and present-day conditions; however, there was a northern range expansion in the continental USA to include parts of Southern Canada in case of *Ae*. *albopictus* in both 2050 and 2070. Future models also anticipated further expansion of *Ae*. *albopictus* to the East to include most of Europe in both time periods. *Aedes aegypti* was anticipated to expand to the South in East Australia in 2050 and 2070. The predictions showed differences in distributional potential of both species between diverse RCPs in 2050 and 2070. Finally, the background similarity test comparing the ENMs of *Ae*. *aegypti* and *Ae*. *albopictus* was unable to reject the null hypothesis of niche similarity between both species (P > 0.05).

**Conclusion:**

These updated maps provided details to better guide surveillance and control programs of *Ae*. *aegypti* and *Ae*. *albopictus*. They have also significant public health importance as a baseline for predicting the emergence of arboviral diseases transmitted by both vectors in new areas across the world.

## Introduction

The yellow fever mosquito *Aedes* (*Stegomyia*) *aegypti* (L.) and the Asian tiger mosquito *Aedes (Stegomyia) albopictus* (Skuse) are two major vectors of several arboviruses [1, 2]. These viruses include dengue virus (DENV), yellow fever virus (YFV), chikungunya virus (CHIKV), and Zika virus (ZIKV) which are widely distributed across tropics and subtropics regions of the world [3–6]. Arboviruses infect millions of cases annually across the world where almost half of the world’s population is at risk of infection with these deadly threats [7–9]. The global emergence of these arboviruses was associated with pathogen, vector, host, and environmental interactions [10, 11]. Previous studies recognized several factors to influence arboviral disease emergence; these factors possibly include further expansion of suitable vector habitats, viral genetic mutations, anthropological behavior, poor sanitary services, commercial transportation, and socioeconomic and land cover changes [2, 11–14]. This rapid emergence and spread of these deadly arboviruses are always associated with the lack of commercially available antivirals or vaccines [6, 15].

*Aedes aegypti* was probably originated in Sub-Saharan Africa from a wild, and zoophilic ancestral species named *Ae*. *aegypti formosus* [16]. Recently, *Ae*. *aegypti* has been introduced and established in much of the tropical and subtropical regions owing to globalization and human activities [2, 17]. *Aedes albopictus* was native to Southeast Asia, islands of Indian Ocean and the western Pacific [18, 19]. Recently, *Ae*. *albopictus* caused public threats by expanding its range to Africa, Europe, and the Americas via human activities and active transportations [19, 20]. *Aedes aegypti* and *Ae*. *albopictus* are invasive and container-breeding mosquitoes [12]. *Aedes aegypti* feeds almost mainly on humans during daylight and rests indoors [21]. *Aedes albopictus* alternatively feeds on humans and animals opportunistically and tends to rest outdoors [22] but has also been shown to exhibit strongly anthropophagic behavior like *Ae*. *aegypti* [19, 20, 23].

The global distributional potential of *Ae*. *aegypti* and *Ae*. *albopictus* was found to be limited by several factors [1, 12]. These factors include climate, socioeconomic factors, and interspecific competition between the two species [1, 12]. Thus, the climatic changes and elevated carbon emissions may drive rapid changes in the global distribution of these vector species and allow several introduction events into new regions of the world. All these changes may trigger the rapid emergence of several arboviral diseases globally [24–26]. So, we still require to better understand the current distributions and possible future shifts of these two species for successful surveillance and control programs of several arboviruses across the world [2]. Here, we updated the global potential distribution of *Ae*. *aegypti* and *Ae*. *albopictus* using the ecological niche modeling approach. We also compared the niches of both species and identified the climate change influences on their global distribution under different emission scenarios in 2050 and 2070.

## Materials and methods

### Occurrence records

We drew all occurrence records for *Ae*. *aegypti* and *Ae*. *albopictus* from the previously published literature [2, 27]. We avoided using the occurrence data from the Global Biodiversity Information Facility (GBIF) and the VectorMap data portal for two reasons; 1) lack of detailed spatial uncertainty associated with each record available via GBIF and VectorMap portals, 2) GBIF duplicated the same dataset retrieved from literature [2, 27]. We downloaded an initial set of 19,929 and 22,137 occurrence records for *Ae*. *aegypti* and *Ae*. *albopictus*, respectively. We reduced these datasets using a variety of quality-control and cleaning steps to reduce any possible bias in calibrating ecological niche models (ENMs). First, we excluded records with higher spatial uncertainty and included only occurrences with exact geographic coordinates in the final dataset. Second, we removed duplicate records, so, only unique records were presented in the final occurrences for each species. The initial set of occurrences consists of 13,991 and 17,280 records with the exact sampling coordinate for *Ae*. *aegypti* and *Ae*. *albobictus*, respectively. We removed duplicate records from this set, so, the data yielded only 4,251 and 3,341 unique records for *Ae*. *aegypti* and *Ae*. *albopictus*, respectively. Finally, we filtered these occurrences based on a distance filter to omit all redundant records occurring in a single 2.5' pixel (≈ 5 km) [28, 29]. We divided randomly the final occurrence dataset for each species into two halves; 50% for model calibration, and 50% for model evaluation.

### Climatic data

To characterize the current global climate, we used bioclimatic data v. 1.4 available from the WorldClim archive (www.worldclim.org). The WorldClim data include 19 bioclimatic variables originally derived from monthly temperature and rainfall values collected from the worldwide weather stations during 1950–2000. We used 2.5ˈ spatial resolution (≈ 5 kilometers) in light of the global extent of our analysis. For future data, we obtained parallel datasets for diverse general circulation models (GCMs) from four representative concentration pathways (RCPs) during 2050 and 2070 to account for possible distributional changes of *Ae*. *aegypti* and *Ae*. *albopictus* under different scenarios and time periods. Representative concentration pathways (RCPs) are scenarios that describe alternative trajectories for CO_2_ emissions and the resulting atmospheric concentration available from the Coupled Model Intercomparison Project Phase 5 (CMIP5); the lowest anthropogenic radiative forcing level scenario RCP 2.6, two median range or stabilization scenarios RCP 4.5 and RCP 6.0, and a comparatively high greenhouse gas emissions RCP 8.5. We used 9 GCMs (S1 File) for each RCP in each time period, with a total of 72 combinations (i.e. 9 GCMs x 4 RCPs x 2 time periods) in four RCPs in 2050 and 2070.

Bioclimatic variables 8–9 and 18–19 were omitted from the analysis, considering the known spatial artifacts in these variables. The remaining of 15 variables were submitted to a principal component analysis (PCA) to reduce the dimensionality and multicollinearity between these variables [28, 29]. The component loadings in the present-day data were used to transform future-climate data using the PCA Projection script written in R software version 3.2.0 [30].

### Ecological niche modeling

We used the maximum entropy algorithm implemented in M_AXENT_ v3.3.3e [31] to predict the distributional potential of *Ae*. *aegypti* and *Ae*. *albopictus* under present-day and future conditions. M_AXENT_ is used to fit species distribution models from occurrence records and environmental variables. All models were based on the first 8 principal components (PCs) described above in the previous analysis; the first 8 PCs summarized more than 99.99% of the overall variance to summarize environmental variation across the world. A crucial step in calibrating the ENMs is determining the calibration area for each species under the analysis [32, 33]. This area is defined as the accessible area “***M***” that have been accessible to the species. We estimated a very broad accessible area “***M***” for each species considering their apparent invasive potential [1, 2]; accessible areas were estimated for both species as much of the world (i.e. between 50.79°N and 60.96°S, and 54.67°N and 41.51°S for *Ae*. *aegypti*, and *Ae*. *albopictus*, respectively).

In M_AXENT_, we used 50% of calibration points for training, with

10 bootstrapped replicates chosen with a random seed. We used M_AXENT_ with clamping and extrapolation deactivated to avoid the risk of over-prediction in non-analogous environments [33].

To summarize the model results under present-day conditions, we used the median values across all runs as an estimate to the ecological niche of each species. For future conditions, we calculated medians across all medians of all single GCMs. The final models were thresholded based on a maximum allowable omission error rate of 5% (E = 5%; [34]), assuming that up to 5% of occurrence data may include errors that misrepresented environmental values. We estimated the uncertainty index of the model predictions via an approach described elsewhere [28]. For present-day conditions, the uncertainty index was derived from the range (maximum—minimum) of predictions in 10 replicate runs in M_AXENT_ (i.e. uncertainty derived from bootstrapped sets of occurrences in M_AXENT_ samples). For the future conditions, an uncertainty index of future model predictions was calculated as the range across all combinations of GCMs in each RCP (i.e. uncertainty derived from the difference in predictions among different GCMs in each RCP).

### Model evaluation

We evaluated the niche models of both species via two approaches; 1) partial receiver operating characteristic (*p*ROC) statistics applied to the 50% subset of occurrences left out before model calibration for testing. We chose *p*ROC as a significance test in light of critiques of the appropriateness of traditional ROC approaches [34, 35]; *p*ROC avoids possible errors raised with traditional ROC provided in M_AXENT_ outputs. *p*ROC statistics were calculated using the PartialROC function available in ENMGadgets package based on 1000 iterations. 2) For further evaluation of model robustness to predict the occurrence of *Ae*. *aegypti* and *Ae*. *albopictus*, we used a set of 2,048 and 2,003 additional occurrence records, respectively. These additional records were obtained from data discarded during early phases of data filtering of the original dataset [2, 27] and ones from WHO Eastern Mediterranean Region [36]. We used a one-tailed cumulative binomial probability test to assess the probability of obtaining the observed level of correct predictions by chance alone given the background expectation of correct predictions determined by the proportional coverage of the study area by regions of predicted suitability.

### Niche overlap of *Ae*. *aegypti* and *Ae*. *albopictus*

We used background similarity test [37] to assess the similarity between the ecological niches of *Ae*. *aegypti* and *Ae*. *albopictus*. We first estimated the accessible area (***M***) for each species in the study [37]; the accessible area for both species was defined as in the previous sections.

To test the null hypothesis of niche similarity between both niches, we used *D*-statistics and Hellinger's *I* implemented in ENMTools [37]. Niche similarity was tested with respect to all environmental variables used to develop the ENM for each species. The background similarity test is based on generating random points from across the accessible area of one species in numbers equal to the numbers of real occurrence data available for that species in the study, with 100 replicate samples, and comparing an ENM based on these “background” points to the ENM of the other species. The null hypothesis of niche similarity was rejected if the ***D*** or ***I*** values fell below the 5^th^ percentile in the random-replicate distribution of similarity values [37]. Finally, we visualized the overlap between both species in 3-dimensional space based on the first three principal components (PCs; the first 3 PCs presents 99.46% of the overall variance) using the software Niche A [38].

## Results

**Fig 1:**
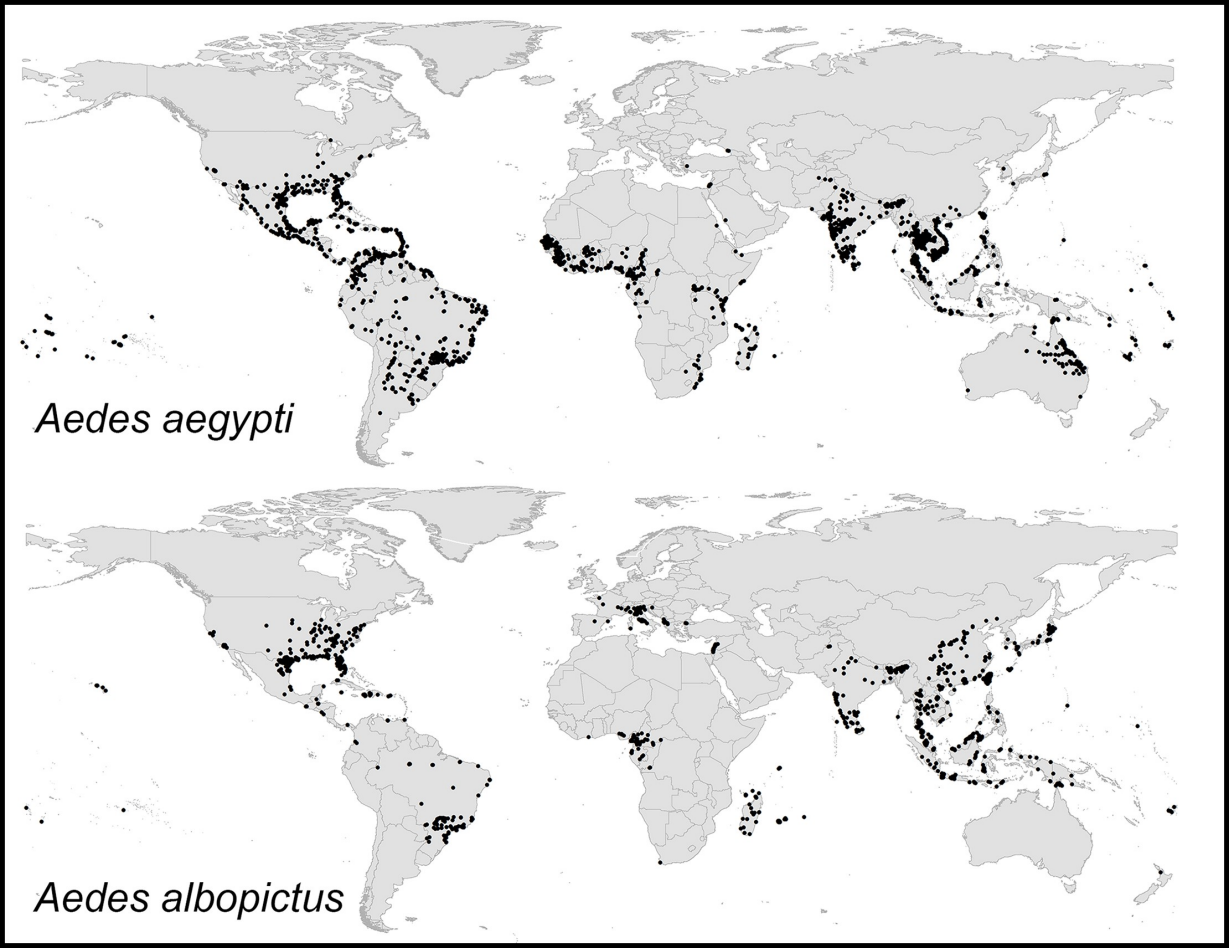
Summary of *Aedes aegypti* and *Ae*. *albopictus* occurrence records available for model calibration and evaluation.

We assembled a total of 2,303 and 1,427 unique occurrence records for *Ae*. *aegypti* and *Ae*. *albopictus*, respectively after detailed cleaning of the initial dataset (Fig 1). Most of the occurrence records are geographically distributed across Asia and Americas; > 55% of *Ae*. *aegypti* records occurred in Brazil, India, Thailand, Mexico, and United States. For *Ae*. *albopictus*, > 67% records occurred only in Taiwan, United States, and Indonesia. The final dataset of occurrences used for analysis is available via online repository available at https://figshare.com/s/6b18c6ce273a3ecaaddc.

**Fig 2:**
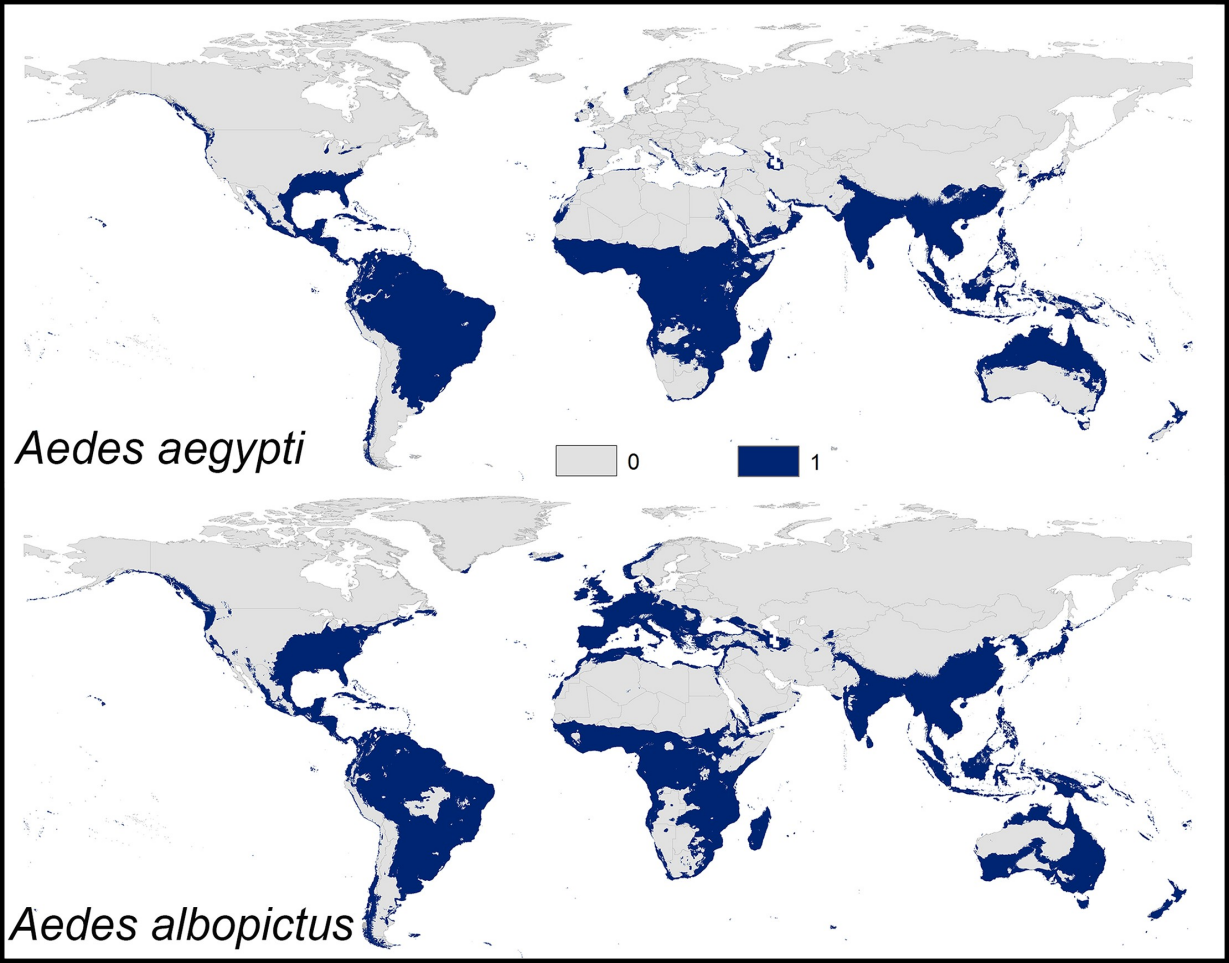
Current potential distribution of *Aedes aegypti* and *Ae*. *albopictus* based on present-day climatic conditions. Navy blue shaded areas were modeled as suitable; gray areas were modeled as unsuitable.

The predicted potential distribution of *Ae*. *aegypti* and *Ae*. *albopictus* coincided considerably with the current and historical known distributions of both species. The geographic distribution of both species coincided in some areas in Asia, and West Africa; however, both species differed markedly in Europe, East Africa, United States, and Australia (Fig 2). *Aedes aegypti* showed a markedly broader distributional potential across tropical and subtropical regions than *Ae*. *albopictus*. Interestingly, *Ae*. *albopictus* was markedly broader in distributional potential across temperate Europe and United States.

In Americas, the distributional potential of *Ae*. *aegypti* was observed in much of South America. The environmentally suitable areas of *Ae*. *aegypti* included the Caribbean islands, most of Southeast USA, and along a narrow zone in the Pacific coasts of Canada and USA. Indeed, *Ae*. *albopictus* showed a broader distribution in the continental USA but narrower distributional potential across South America (Fig 2); *Ae*. *albopictus* broadly extended from Southeast to North USA and South Canadian border.

In Europe, *Ae*. *aegypti* was predicted to occur in a limited narrow zone along the Mediterranean coast of Turkey, Greece, Cyprus, Croatia, Albania, Italy, Spain, France, and the Atlantic coasts of Portugal. Interestingly, all Spanish islands in the Atlantic Ocean were environmentally suitable for *Ae*. *aegypti* occurrence (S2 File). *Ae*. *albopictus* showed expanded distributional potential across much of Western Europe and the Balkan region.

In Africa, *Ae*. *aegypti* was widely distributed across Sub-Saharan countries; however, its distributional potential is very limited in Namibia, Botswana, South Africa, Angola, and Zambia. Indeed, *Ae*. *aegypti* was also predicted across West Morocco and Western Sahara, North Algeria and Tunisia, and across the Red Sea coast in Egypt and Sudan (S3 File). The distributional potential of *Ae*. *albopictus* was markedly narrower across East Africa; however, the species occurred across the Mediterranean coast extending from Morocco to Egypt (S4 File). Indeed, environmentally suitable conditions of *Ae*. *albopictus* were identified also on the Red Sea coast in Egypt (S5 File).

In Asia, *Ae*. *aegypti* showed distributional patterns across much of the continent including Central and Southern Asia. The predicted distributional potential of *Ae*. *aegypti* included also much of Oceania, Northern Australia, Eastern and Western coasts of Australia and New Zealand. The species also occurred in Syria, Lebanon, Israel, Western Saudi Arabia, and the Western coasts of the Arabian Gulf. *Ae*. *albopictus* showed similar distributional patterns in Asia; however, it showed a marked increase in suitable areas across Lebanon, Israel, the Mediterranean coasts of Syria but narrower suitable areas in Saudi Arabia, and Southwestern Yemen. The distributional range of *Ae*. *albopictus* was narrower in North but broader in East and South Australia.

ENMs for *Ae*. *aegypti* and *Ae*. *albopictus* yielded predictions that gave area under the curve (AUC) ratios above the null expectations in partial ROC analyses of *Ae*. *aegypti* and *Ae*. *albopictus* (P < 0.001; Table 1). ENMs successfully anticipated 98% (1,999/2,048) and 99% (1,985/2,003) of additional independent records for both *Ae*. *aegypti* and *Ae*. *albopictus*, respectively, which is statistically better than random expectations (P < 0.001; S6 File).

**Table 1:** Partial area under the curve (AUC) ratios summarizing evaluations of ecological niche models of arboviral vectors *Aedes aegypti* and *Ae*. *albopictus* based on 1000 bootstrap iterations. no. NS indicates the number out of 1000 random replicate analyses for which AUC ratio was greater than 1.

Species	no. NS	*p*ROC ratio			
		Minimum	Maximum	Mean	Median
*Aedes aegypti*	1000	1.33	1.47	1.39	1.39
*Aedes albopictus*	1000	1.35	1.60	1.46	1.46

**Fig 3:**
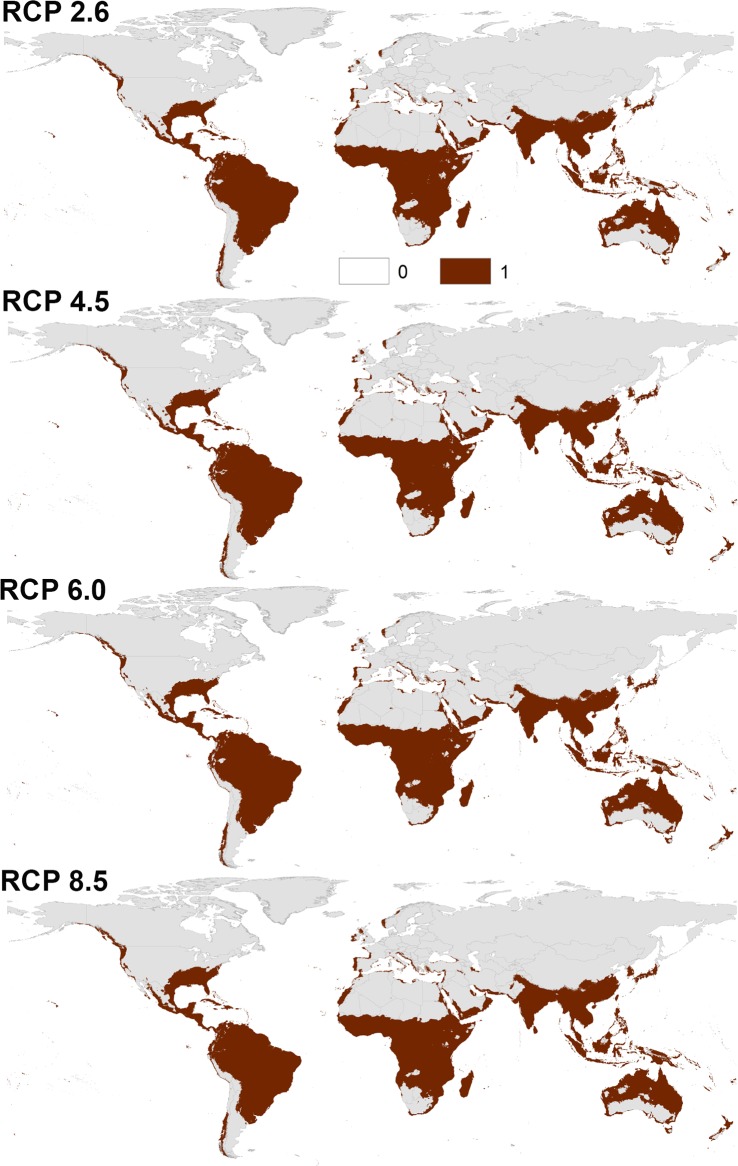
Predicted future potential distribution of *Aedes aegypti* under four future representative concentration pathways of climate conditions in 2050. Brown areas are modeled suitable conditions; gray areas are unsuitable conditions.

**Fig 4:**
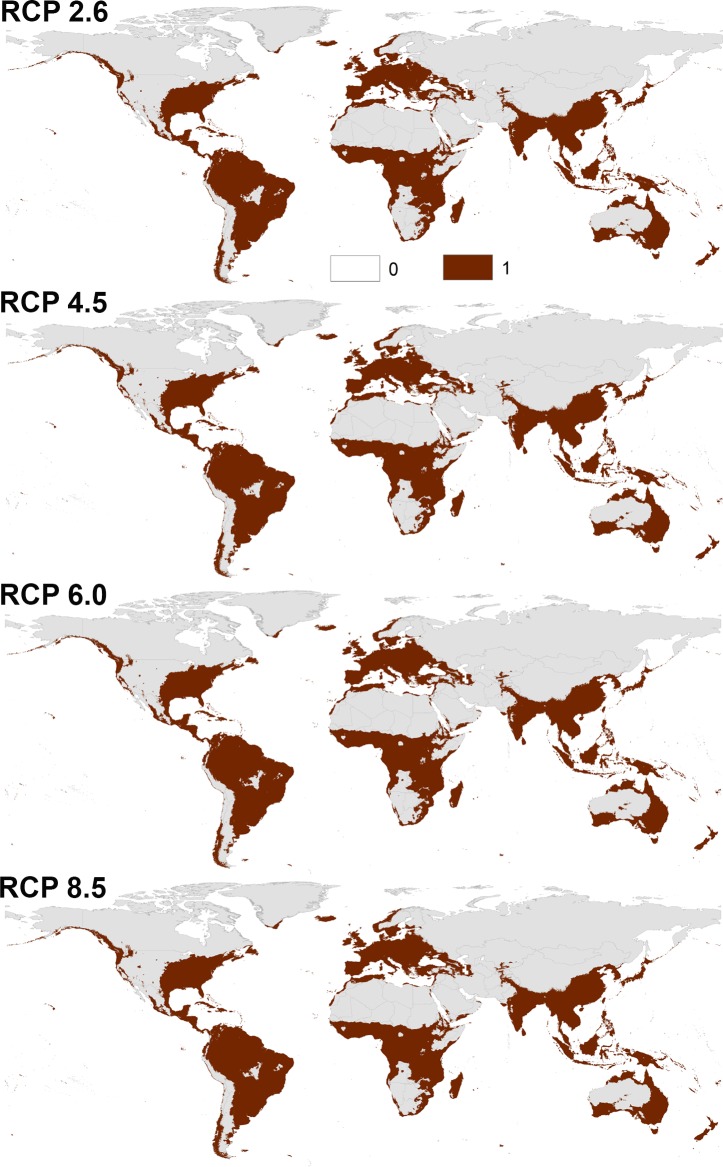
Predicted future potential distribution of *Aedes albopictus* under four future representative concentration pathways of climate conditions in 2050. Brown areas are modeled suitable conditions; gray areas are unsuitable conditions.

Transferring the calibrated models of both species to the future showed a similarity between the overall distributional patterns of future-day and present-day conditions (Figs 3 and 4, S7 and S8 Files); however, there were few differences in the predictions. Indeed, a northern range expansion was observed in continental USA for both species and expanded further to include parts of Southern Canada in case of *Ae*. *albopictus* in both 2050 and 2070 (Fig 4, S8 File). We anticipated further expansion of *Ae*. *albopictus* to the East to include most Europe in both time periods. *Aedes aegypti* was anticipated to expand to the South in East Australia in 2050 and 2070.

The predictions showed differences between diverse RCPs in 2050 and 2070 (Figs 3 and 4, S7 and S8 Files); both species is anticipated to increase from RCP 2.6 to RCP 8.5 and from 2050 to 2070. Between the present-day and 2050, the potential distribution area of *Ae*. *aegypti* increased by 1.89%, 2.38%, 2.60%, and 3.02% under RCP 2.6, RCP 4.5, RCP 6.0, and RCP 8.5, respectively. Between the present day and 2070, the potential distributional area of *Ae*. *aegypti* was anticipated to increase by 2.25%, 2.84%, 3.03%, and 4.47% under RCP 2.6, RCP 4.5, RCP 6.0, and RCP 8.5, respectively. For *Ae*. *albopictus*, the overall distributional potential was anticipated to increase by 1.56%, 2.24%, 2.30%, 2.70% under RCP 2.6, RCP 4.5, RCP 6.0, and RCP 8.5, respectively between the present-day and 2050. Between the present-day and 2070, the potential distribution area of *Ae*. *albopictus* increased by 2.17%, 2.88%, 3.01%, and 4.21% under RCP 2.6, RCP 4.5, RCP 6.0, and RCP 8.5, respectively. We provided detailed maps of potential distributions under each individual GCM via the supplementary materials; the final future projections are summarized as a GeoTIFF data file at https://figshare.com/s/6b18c6ce273a3ecaaddc.

**Fig 5:**
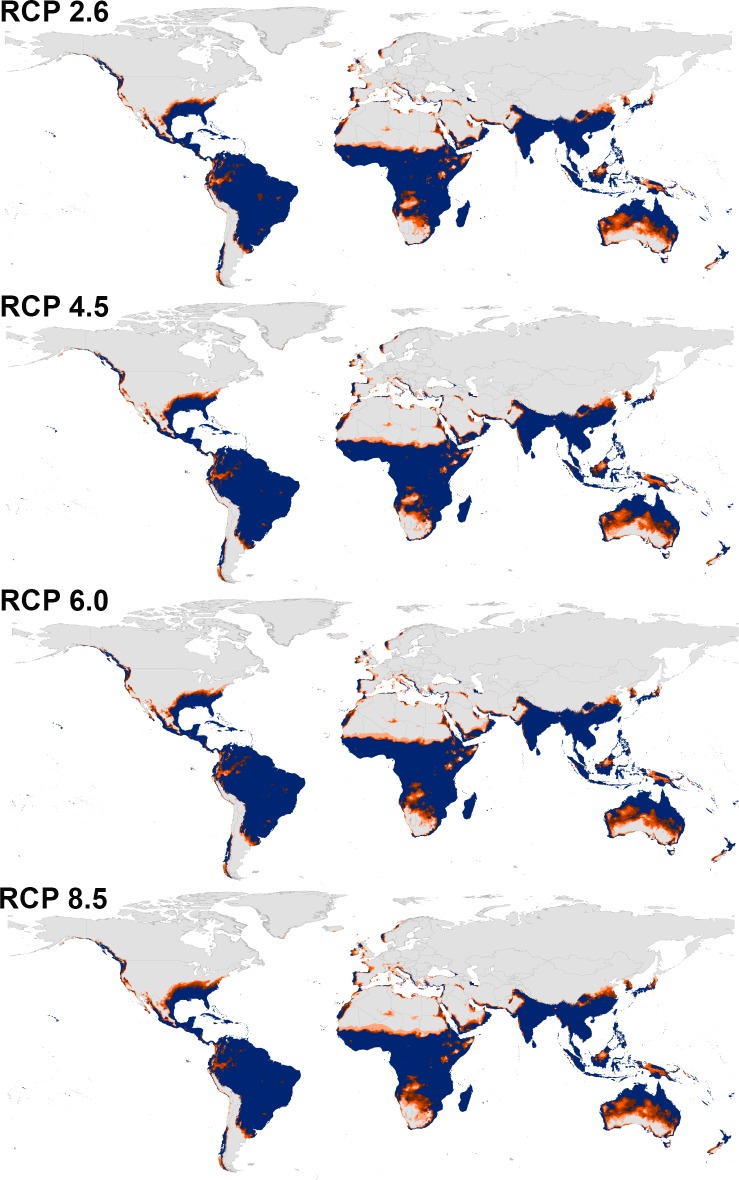
Summary of the modeled global distribution of *Aedes aegypti* under both current and future climatic conditions in 2050 showing stability of predictions at present and into the future, and to illustrate differences among representative concentration pathways (RCPs). Navy blue represents model stability under both current and future conditions, dark orange represents agreement among all climate models in anticipating the potential distributional areas in the future, and light orange indicates low agreement between diverse models as regards distributional potential in the future.

**Fig 6:**
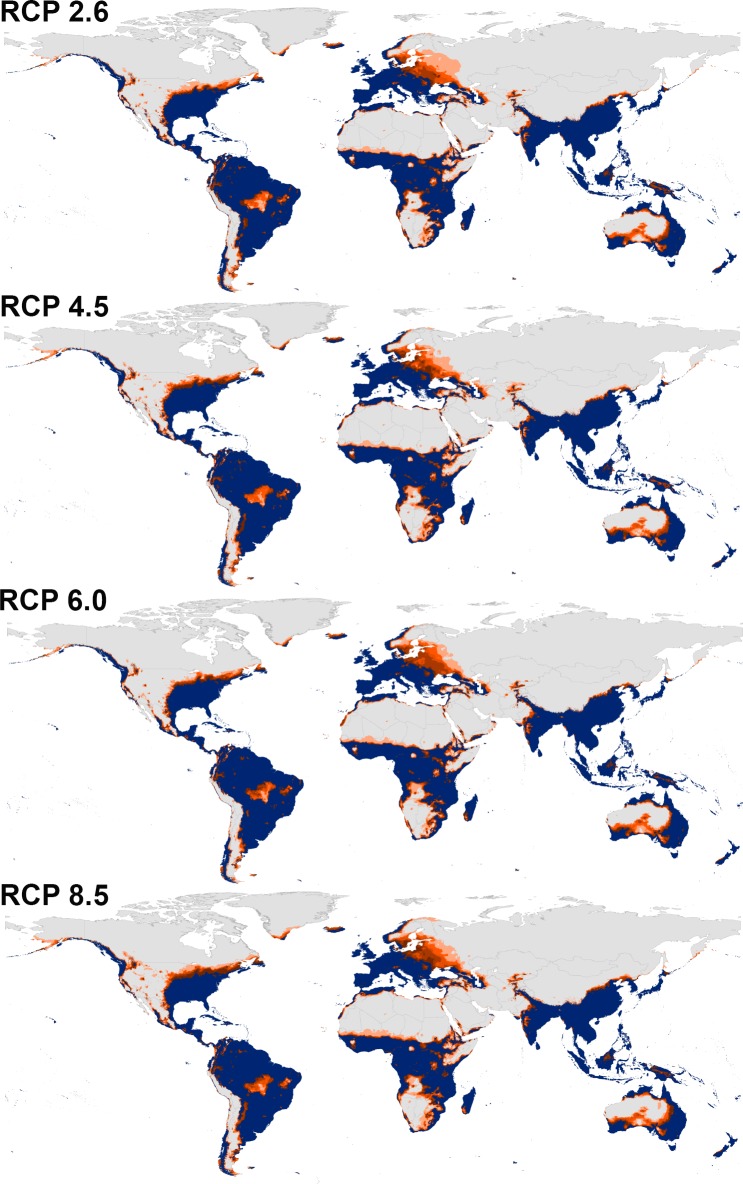
Summary of the modeled global distribution of *Aedes albopictus* under both current and future climatic conditions in 2050 showing stability of predictions at present and into the future, and to illustrate differences among representative concentration pathways (RCPs). Navy blue represents model stability under both current and future conditions, dark orange represents agreement among all climate models in anticipating the potential distributional areas in the future, and light orange indicates low agreement between diverse models as regards distributional potential in the future.

Detailed maps of model stability for both species illustrated differences among diverse RCPs in 2050, and 2070 (Figs 5 and 6; S7 and S8 Files). For *Ae*. *aegypti*, the highest stability of the models among present-day and future conditions appeared in the belt between 37°S and 35°N, which include South America, Southern USA, Sub-Saharan Africa, Red Sea coast, and a narrow zone in West and North Africa, South Asia, and North Australia (Fig 5; S9 File). Areas with higher agreement among diverse GCMs included parts of Northern South America, Northern USA, Western Africa, Western Saudi Arabia, and Eastern China.

*Aedes albopictus* showed high agreement between present-day and future conditions in South America, Eastern USA, Western Europe, Central and South Asia; however, Northern USA, and Eastern Europe were characterized as areas with higher agreement among all GCMs in both time periods (Fig 6; S10 File).

Under present-day conditions, both species showed variation in uncertainty index among different regions (S11 File). *Aedes aegypti* showed higher uncertainty in parts of Southern coast of US, East Africa, parts of Southeast Asia whereas low uncertainty was observed in North Africa, Sub-Saharan Africa, Central and South America, and Central and North America (S11 File). For *Ae*. *albopictus*, higher uncertainty was observed in North Africa, West Africa, West of the Red Sea coast, narrow zones in Europe and North America. Lower uncertainty was observed in North America, most continental Africa, Asia, and Europe.

Under future conditions, the level of uncertainty varied among different time periods and diverse RCPs for both species. Highest variation in model predictions for *Ae*. *aegypti* across all RCPs was observed in Eastern China, Southern USA, Northern South America. *Aedes albopictus* also showed high variation at diverse places across the Americas, parts of Europe, and Eastern Asia.

**Fig 7:**
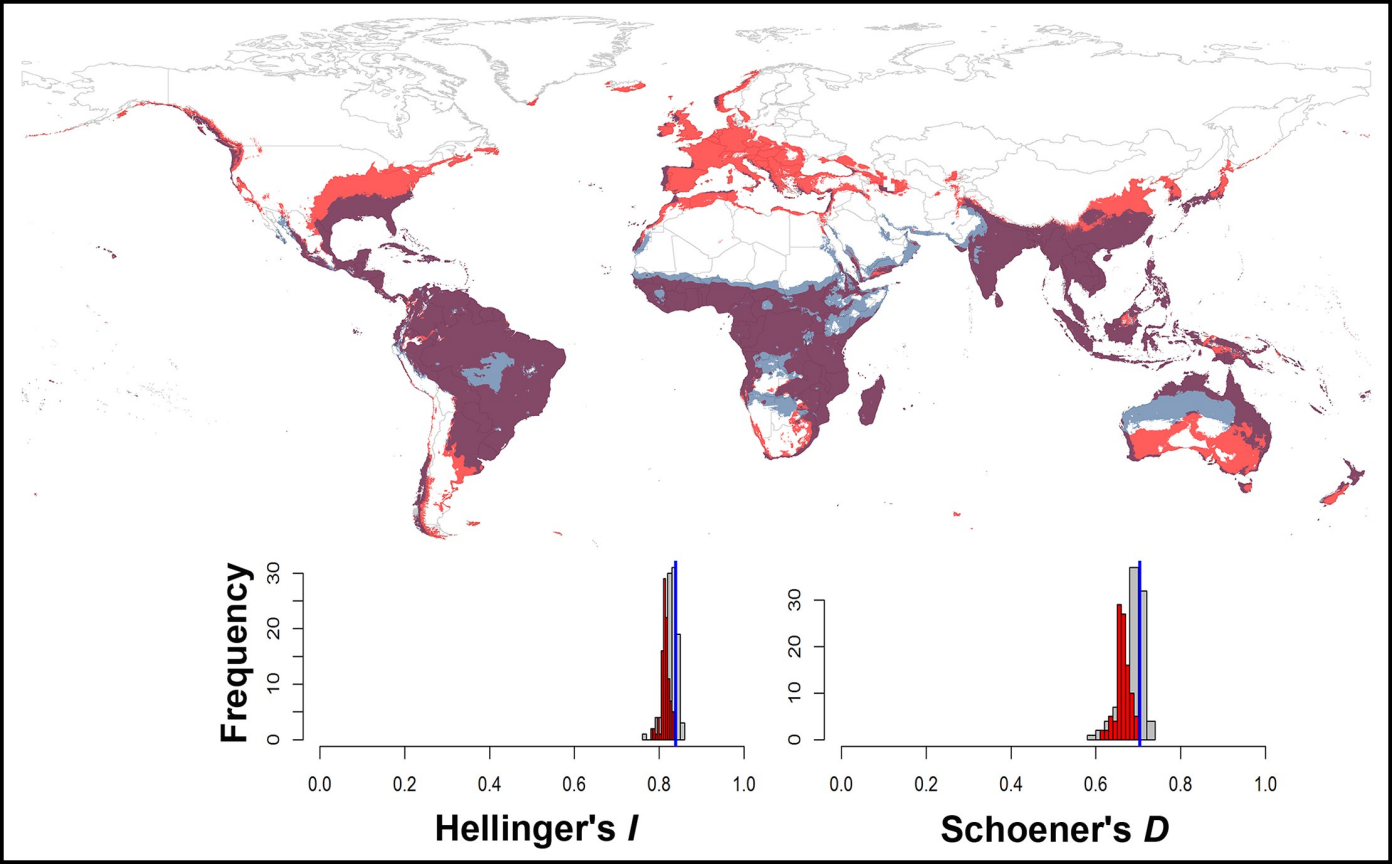
Background similarity test showing overall niche overlap between ecological niche models for *Aedes aegypti* and *Ae*. *albopictus*. The vertical blue line shows observed niche overlap, and the histograms show the distribution of the background similarity values among 100 random replicates, for the *I* and *D* similarity metrics. On the maps, dark gray and light red shading indicates the modeled suitable areas for *Ae*. *aegypti* and *Ae*. *albopictus*, respectively; dark purple shading shows areas of overlap between the two species.

**Fig 8:**
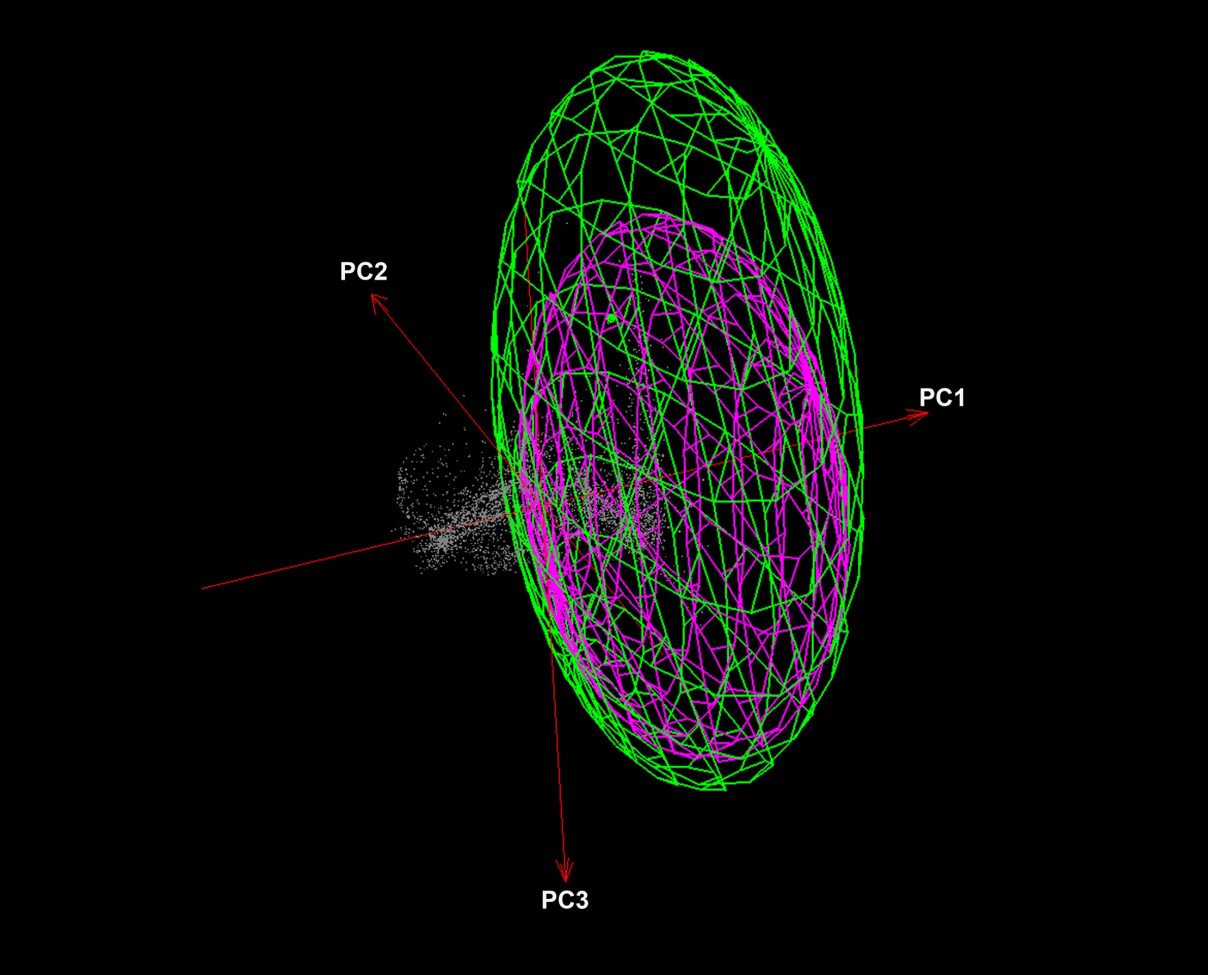
Visualization of ecological niches of *Aedes aegypti*, and *Ae*. *albopictus* in three environmental dimensions (PC1, PC2, and PC3). Niches are represented as minimum volume ellipsoids to illustrate the limits under which the species has been sampled. Gray shading represents environmental background, green ellipsoid represents *Aedes aegypti*, and pink is *Aedes albopictus*.

The background similarity test comparing the ENMs of *Ae*. *aegypti* and *Ae*. *albopictus* was unable to reject the null hypothesis of niche similarity between both species (P > 0.05; Fig 7). We used NicheA to visualize overall overlap between the two species based on three dimensions of PCs (Fig 8), which revealed broad overlap in environmental conditions used by both species.

## Discussion

The present study provided the most updated and detailed maps of the current global potential distributions of two arboviral vectors, *Ae*. *aegypti* and *Ae*. *albopictus*, and anticipated their potential distributions under changing climate. These maps provided significant public health importance for several reasons; 1) they estimate probabilities of occurrences for two primary vectors of major arboviruses that are increasingly spread across the world, 2) they examine the possible climate change influences on the distributional potential of key disease vectors, and 3) they provide a primary source for maps used to prioritize surveillance and control programs of both vectors and *Aedes*-borne diseases.

Two major studies previously identified the global distributional potential of *Ae*. *aegypti* and *Ae*. *albopictus* [1, 2]. One of these studies recognized lack of occurrence data and significantly underestimated the potential distribution of these species [1]. The rest of studies used a similar set of occurrences; however, these occurrences have duplicated records, and others that are of uncertainty greater than pixel size (i.e. scale mismatch between sampling sites and spatial resolution of climatic variables); correction of these problems should lead to higher model performance [39, 40]. Here, we updated the ecological niches of *Ae*. *aegypti* and *Ae*. *albopictus* based on thinned and unbiased occurrence records with the exact sampling localities; we cleaned occurrences and used the unique records that presented the accurate sampling sites. Our ENMs improved the predictions of both species; these ENMs are statistically robust and predicted most additional independent records for both species. Interestingly, these models predicted about 98% and 99% of additional records of *Ae*. *aegypti*, and *Ae*. *albopictus*, respectively. The presence of these species were recently confirmed via active surveillance of these two vectors in the same regions anticipated by our models [41–44].

Our models were the first to look more closely for the distributional patterns of *Ae*. *aegypti* in Spanish islands, Chile, and the Red Sea coast in Egypt and Sudan where recent outbreaks of arboviral diseases were reported [44]. Recent surveillance of *Ae*. *aegypti* suggested known presences of this species in almost all of these regions [44, 45]. *Aedes albopictus* model concurs with recent surveillance of this invasive species across the world [41, 43]; our models anticipated new areas in North Africa, Canada (e.g., Ontario), and United Kingdom where the species was also identified [42, 43, 46]. Here, the study added another dimension of novelty by using the most updated version of climatic scenarios rather than using the previous version of future climatic models as implemented in the previous study [1].

The results of our models overlap in most cases with the previously published models by Kraemer and colleagues [2]; however, our models recognized different distributional patterns to the one predicted by Campbell *et al*. [1]. The latter study [1] underestimated occurrences of both species in many areas where both species are abundant [18, 27, 42–45]. For example, Campbell and colleagues [1] underestimated *Ae*. *aegypti* in South Western Africa, Arabian Peninsula, India, and China and *Ae*. *albopictus* in Europe, North Africa, Australia, and Eastern Brazil. Surprisingly, our models added some additional sites to the ones predicted by these previous studies [1, 2]. In this study, an additional distributional potential of *Ae*. *aegypti* was observed in other regions including New Zealand, Oman, Yemen, North Africa, Western Sahara, Northern coasts of Spain, Western South of France, Mediterranean coasts of Turkey, Chile, and the Western coasts of Canada. Indeed, *Ae*. *aegypti* was sampled previously from some of these sites [42, 45, 47, 48]. For *Ae*. *albopictus* models, we still see incongruences between our models and the ones published previously in regards to the environmental suitability predicted by our model in several regions in New Zealand, Southern Australia, Yemen, Western Saudi Arabia, Africa, Western Europe, and Chile; all previous models [1, 2] underestimated the distributional potential of *Ae*. *albopictus* in Europe. These previous studies failed to better estimate the distributional potential of both species owing to either lack of occurrence data [1] or biased dataset with associated uncertainties of occurrences [2].

The population dynamics and distribution potential of *Ae*. *Aegypti* and *Ae*. *albopictus* proved to respond positively to elevated temperature [19, 49]. Indeed, both species revealed the potential for range expansion under changing climate. The latter may trigger the invasion of those species to new habitats where climate and resources allow the establishment of new populations of the two species [50]. *Aedes aegypti* and *Ae*. *albopictus* were anticipated to invade new sites or re-emerge in other sites, for example, the ranges of both species were predicted to expand further to include Canada, other sites in Europe, and parts of the Middle East and North Africa. These distributional patterns coincided with the patterns observed previously for the same vector species [12, 18, 27, 42–45].

The global distribution of *Ae*. *aegypti* and *Ae*. *albopictus* were proved to be influenced mainly by the climatic conditions [1, 2]. Therefore, under the current and future climatic conditions, the distribution patterns of both species was shown to be concentrated in the tropical and subtropical regions, with a dominant distribution of *Ae*. *albopictus* over the temperate regions whereas *Ae*. *aegypti* showed wider distribution over the tropical and subtropical regions (Fig 2). Although weather and climatic factors are important drivers for establishing the population of *Ae*. *aegypti* as well as *Ae*. *albopictus* [49, 51], these mosquitoes also depend on the presence of water containers for oviposition and larval development. This concurs with human-related activities in rainfall periods or drought periods where lack of water resources is predominant [12]. These human activities are important drivers of mosquito population dynamics by adding new breeding sites via water storage in tanks.

*Aedes albopictus* showed notable suitability over *Ae*. *aegypti* in temperate climate (Fig 2) concurring with species occurrence in the temperate regions, the same result was anticipated by a previous study [2]. The climatic factors in temperate regions are significantly different from the characteristic tropical and subtropical climate where the two species occurred. The latter suggested the occurrence of other factors affecting the distribution of the two species; these factors may include biotic factors, for example, possible interspecific competition between both species under the study.

Interestingly, both species under the analyses might occupy similar ecological niches (Figs 7 and 8). The latter finding is parallel with several other studies that recognized *Ae*. *aegypti* and *Ae*. *albopictus* as sympatric species; both species tend to breed in similar habitats [52, 53]. Competitive displacement is possibly occurred between *Ae*. *aegypti* and *Ae*. *albopictus*, for example, *Ae*. *albopictus* has the competitive advantage under local field conditions in North American populations [54]. The latter possibly contributed to the displacement of *Ae*. *aegypti* in some localities of the continental USA, possibly after the invasion of *Ae*. *albopictus*. This pattern could be indistinguishable to what occurred in Europe; *Ae*. *aegypti* populations in Europe may be displaced by *Ae*. *albopictus* owing to the interspecific competition. This displacement may be related to several factors [22, 54–56]. First, *Ae*. *albopictus* maintains a strong ecological and physiological plasticity that allows for its strong capacity to adapt rapidly to wide varieties of habitats [22, 55]; this adaptation may include cold temperatures where the species becomes dormant during the winter of temperate regions [22, 55]. Second, the cross-insemination occurred naturally between *Ae*. *aegypti* and *Ae*. *albopictus* and the sterilizing effects of male accessory gland products [54, 56]. The latter favors *Ae*. *albopictus* in interspecific mating and support satyrization which presumably explain the rapid displacements and fitness reduction owing to drastic reproductive loss of *Ae*. *aegypti* females satyrized by *Ae*. *albopictus* males [54, 56].

This study updated the potential distribution of both species; however, it identified some limitations; 1) absence of human-based variables in model calibration, and 2) models didn’t consider the component of interspecific competition between *Ae*. *aegypti* and *Ae*. *albopictus*. Johnson *et al*. [50] excluded human population as a predictor in their model considering that even rural areas typically have a population that able to support *Ae*. *aegypti* under favorable climatic conditions. Alternatively, another study identified human population as a key factor to shape the ecological niche of at least *Ae*. *aegypti* [57]. The inclusion of these factors to the model will allow better predictions; however, lack of parallel future data remains a gap in knowledge, particularly when we project these models to the future.

In summary, our study improved the distributional potential of *Ae*. *aegypti* and *Ae*. *albopictus* and placed several other regions at risk of vector invasions. The latter may trigger emergence and re-emergence of several diseases to new and historical sites, respectively. These diseases included ones of major public health concerns, particularly dengue, chikungunya, as well as Zika. The invasion of these species placed several new areas at risk of these deadly diseases, particularly in the Middle East [36].

## Supporting information

S1 File List of general circulation models used in the ecological niche modeling analyses of *Aedes aegypti* and *Ae. albopictus* under future climatic conditions. (PDF)

S2 File Current potential distribution of *Aedes aegypti* in Spanish islands based on present-day conditions. Navy blue shaded areas were modeled as suitable; gray areas were modeled as unsuitable.
(PDF)

S3 File Current potential distribution of *Aedes aegypti* in Sudan, and Red Sea coasts in Egypt and Saudi Arabia based on present-day conditions. Navy blue shaded areas were modeled as suitable; gray areas were modeled as unsuitable.
(PDF)

S4 File Current potential distribution of *Aedes albopictus* in North Africa based on present-day conditions. Navy blue shaded areas were modeled as suitable; gray areas were modeled as unsuitable.
(PDF)

S5 File Current potential distribution of *Aedes albopictus* in Red Sea coasts based on present-day conditions. Navy blue shaded areas were modeled as suitable; gray areas were modeled as unsuitable.
(PDF)

S6 File Relationship of additional independent records of *Ae. aegypti* and *Ae. albopictus* to areas predicted as suitable for *Ae. aegypti* and *Ae. albopictus*, respectively. Yellow points are independent occurrence data from the Old World and North America. Blue areas are represented as suitable and gray as unsuitable.
(PDF)

S7 File Predicted future potential distribution of *Aedes aegypti* under four future representative concentration pathways of climate conditions in 2070. Brown areas are modeled suitable conditions; gray areas are unsuitable conditions.
(PDF)

S8 File Predicted future potential distribution of *Aedes albopictus* under four future representative concentration pathways of climate conditions in 2070. Brown areas are modeled suitable conditions; gray areas are unsuitable conditions.
(PDF)

S9 File Summary of the modeled global distribution of *Aedes aegypti* under both current and future climatic conditions in 2070 showing stability of predictions at present and into the future, and to illustrate differences among representative concentration pathways (RCPs). Navy blue represents model stability under both current and future conditions, dark orange represents agreement among all climate models in anticipating the potential distributional areas in the future, and light orange indicates low agreement between diverse models as regards distributional potential in the future.
(PDF)

S10 File Summary of the modeled global distribution of *Aedes albopictus* under both current and future climatic conditions in 2070 showing stability of predictions at present and into the future, and to illustrate differences among representative concentration pathways (RCPs). Navy blue represents model stability under both current and future conditions, dark orange represents agreement among all climate models in anticipating the potential distributional areas in the future, and light orange indicates low agreement between diverse models as regards distributional potential in the future.
(PDF)

S11 File Visualization of uncertainty estimates associated with present-day and future predictions of *Aedes aegypti* and *Ae. albopictus*. (PDF)

## References

[1] Campbell LP, Luther C, Moo-Llanes D, Ramsey JM, Danis-Lozano R, Peterson AT. Climate change influences on global distributions of dengue and chikungunya virus vectors. Philos Trans R Soc London [Biol]. 2015;370(1665). 10.1098/rstb.2014.0135 PMC4342968 25688023

[2] Kraemer MU, Sinka ME, Duda KA, Mylne AQ, Shearer FM, Barker CM, et al The global distribution of the arbovirus vectors *Aedes aegypti* and *Ae. albopictus*. eLife. 2015;4:e08347 10.7554/eLife.08347 26126267 PMC4493616

[3] Jentes ES, Poumerol G, Gershman MD, Hill DR, Lemarchand J, Lewis RF, et al The revised global yellow fever risk map and recommendations for vaccination, 2010: consensus of the Informal WHO Working Group on Geographic Risk for Yellow Fever. Lancet Infect Dis. 2011;11(8):622–32. 10.1016/S1473-3099(11)70147-5 21798462

[4] Leparc-Goffart I, Nougairede A, Cassadou S, Prat C, de Lamballerie X. Chikungunya in the Americas. Lancet. 2014;383(9916):514 10.1016/S0140-6736(14)60185-9 24506907

[5] Samy AM, Thomas SM, Wahed AA, Cohoon KP, Peterson AT. Mapping the global geographic potential of Zika virus spread. Mem Inst Oswaldo Cruz. 2016;111(9):559–60. 10.1590/0074-02760160149 27653360 PMC5027865

[6] Simmons CP, Farrar JJ, Nguyen v V, Wills B. Dengue. N Engl J Med. 2012;366(15):1423–32. 10.1056/NEJMra1110265 22494122

[7] Morrison AC, Gray K, Getis A, Astete H, Sihuincha M, Focks D, et al Temporal and geographic patterns of *Aedes aegypti* (Diptera: Culicidae) production in Iquitos, Peru. J Med Entomol. 2004;41(6):1123–42. 15605653 10.1603/0022-2585-41.6.1123

[8] Bhatt S, Gething PW, Brady OJ, Messina JP, Farlow AW, Moyes CL, et al The global distribution and burden of dengue. Nature. 2013;496(7446):504–7. 10.1038/nature12060 23563266 PMC3651993

[9] Johansson MA, Powers AM, Pesik N, Cohen NJ, Staples JE. Nowcasting the spread of chikungunya virus in the Americas. PloS one. 2014;9(8):e104915 10.1371/journal.pone.0104915 25111394 PMC4128737

[10] Bogoch II, Creatore MI, Cetron MS, Brownstein JS, Pesik N, Miniota J, et al Assessment of the potential for international dissemination of Ebola virus via commercial air travel during the 2014 west African outbreak. Lancet. 2015;385(9962):29–35. 10.1016/S0140-6736(14)61828-6 25458732 PMC4286618

[11] Engering A, Hogerwerf L, Slingenbergh J. Pathogen-host-environment interplay and disease emergence. Emerg Microbes Infect. 2013;2(2):e5 10.1038/emi.2013.5 26038452 PMC3630490

[12] Eisen L, Moore CG. *Aedes* (*Stegomyia*) *aegypti* in the continental United States: a vector at the cool margin of its geographic range. J Med Entomol. 2013;50(3):467–78. 23802440 10.1603/me12245

[13] Gandon S, Hochberg ME, Holt RD, Day T. What limits the evolutionary emergence of pathogens? Philos Trans R Soc London [Biol]. 2013;368(1610):20120086. 10.1098/rstb.2012.0086 PMC3538453 23209168

[14] Liang G, Gao X, Gould EA. Factors responsible for the emergence of arboviruses; strategies, challenges and limitations for their control. Emerg Microbes Infect. 2015;4(3):e18 10.1038/emi.2015.18 26038768 PMC4395659

[15] Roy CJ, Adams AP, Wang E, Plante K, Gorchakov R, Seymour RL, et al Chikungunya vaccine candidate is highly attenuated and protects nonhuman primates against telemetrically monitored disease following a single dose. J Infect Dis. 2014;209(12):1891–9. 10.1093/infdis/jiu014 24403555 PMC4038141

[16] Brown JE, McBride CS, Johnson P, Ritchie S, Paupy C, Bossin H, et al Worldwide patterns of genetic differentiation imply multiple 'domestications' of *Aedes aegypti*, a major vector of human diseases. Proc Biol Sci. 2011;278(1717):2446–54. 10.1098/rspb.2010.2469 21227970 PMC3125627

[17] Brown JE, Evans BR, Zheng W, Obas V, Barrera-Martinez L, Egizi A, et al Human impacts have shaped historical and recent evolution in *Aedes aegypti*, the dengue and yellow fever mosquito. Evol. 2014;68(2):514–25. 10.1111/evo.12281 PMC3946797 24111703

[18] Izri A, Bitam I, Charrel RN. First entomological documentation of *Aedes* (*Stegomyia*) *albopictus* (Skuse, 1894) in Algeria. Clin Microbiol Infect. 2011;17(7):1116–8. 10.1111/j.1469-0691.2010.03443.x 21435096

[19] Delatte H, Gimonneau G, Triboire A, Fontenille D. Influence of temperature on immature development, survival, longevity, fecundity, and gonotrophic cycles of *Aedes albopictus*, vector of chikungunya and dengue in the Indian Ocean. J Med Entomol. 2009;46(1):33–41. 19198515 10.1603/033.046.0105

[20] Ponlawat A, Harrington LC. Blood feeding patterns of *Aedes aegypti* and *Aedes albopictus* in Thailand. J Med Entomol. 2005;42(5):844–9. 16363170 10.1093/jmedent/42.5.844

[21] Scott TW, Takken W. Feeding strategies of anthropophilic mosquitoes result in increased risk of pathogen transmission. Trends Parasitol. 2012;28(3):114–21. 10.1016/j.pt.2012.01.001 22300806

[22] Paupy C, Delatte H, Bagny L, Corbel V, Fontenille D. *Aedes albopictus*, an arbovirus vector: from the darkness to the light. Microbes Infect. 2009;11(14–15):1177–85. 10.1016/j.micinf.2009.05.005 19450706

[23] Delatte H, Desvars A, Bouetard A, Bord S, Gimonneau G, Vourc'h G, et al Blood-feeding behavior of *Aedes albopictus*, a vector of Chikungunya on La Reunion. Vector Borne Zoonotic Dis. 2010;10(3):249–58. 10.1089/vbz.2009.0026 19589060

[24] Carrington LB, Simmons CP. Human to mosquito transmission of dengue viruses. Front Immunol. 2014;5:290 10.3389/fimmu.2014.00290 24987394 PMC4060056

[25] Messina JP, Brady OJ, Pigott DM, Golding N, Kraemer MU, Scott TW, et al The many projected futures of dengue. Nat Rev Microbiol. 2015;13(4):230–9. 10.1038/nrmicro3430 25730702

[26] Semenza JC, Sudre B, Miniota J, Rossi M, Hu W, Kossowsky D, et al International dispersal of dengue through air travel: importation risk for Europe. PLoS Negl Trop Dis. 2014;8(12):e3278 10.1371/journal.pntd.0003278 25474491 PMC4256202

[27] Kraemer MU, Sinka ME, Duda KA, Mylne A, Shearer FM, Brady OJ, et al The global compendium of *Aedes aegypti* and *Ae. albopictus* occurrence. Scientific data. 2015;2:150035 10.1038/sdata.2015.35 26175912 PMC4493829

[28] Samy AM, Elaagip AH, Kenawy MA, Ayres CF, Peterson AT, Soliman DE. Climate Change Influences on the Global Potential Distribution of the Mosquito *Culex quinquefasciatus*, Vector of West Nile Virus and Lymphatic Filariasis. PloS one. 2016;11(10):e0163863 10.1371/journal.pone.0163863 27695107 PMC5047650

[29] Samy AM, Peterson AT. Climate Change Influences on the Global Potential Distribution of Bluetongue Virus. PloS one. 2016;11(3):e0150489 10.1371/journal.pone.0150489 26959424 PMC4784974

[30] R Development Core Team. A Language and Environment for Statistical Computing. R Foundation for Statistical Computing, Vienna, Austia 2015 Available at http://www.R-project.org.

[31] Phillips SJ, Anderson RP, Schapire RE. Maximum entropy modeling of species geographic distributions. Ecol Model. 2006;190(3):231–59.

[32] Barve N, Barve V, Jiménez-Valverde A, Lira-Noriega A, Maher SP, Peterson AT, et al The crucial role of the accessible area in ecological niche modeling and species distribution modeling. Ecol Model. 2011;222(11):1810–9.

[33] Owens HL, Campbell LP, Dornak LL, Saupe EE, Barve N, Soberón J, et al Constraints on interpretation of ecological niche models by limited environmental ranges on calibration areas. Ecol Model. 2013;263:10–8.

[34] Peterson AT, Papeş M, Soberón J. Rethinking receiver operating characteristic analysis applications in ecological niche modeling. Ecol Model. 2008;213(1):63–72.

[35] Lobo JM, Jiménez-Valverde A, Real R. AUC: a misleading measure of the performance of predictive distribution models. Glob Ecol Biogeogr. 2008;17(2):145–51.

[36] Ducheyne E, Tran Minh NN, Haddad N, Bryssinckx W, Buliva E, Simard F, et al Current and future distribution of *Aedes aegypti* and *Aedes albopictus* (Diptera: Culicidae) in WHO Eastern Mediterranean Region. Int J Health Geogr. 2018;17(1):4 10.1186/s12942-018-0125-0 29444675 PMC5813415

[37] Warren DL, Glor RE, Turelli M. ENMTools: a toolbox for comparative studies of environmental niche models. Ecography. 2010;33(3):607–11.

[38] Qiao H, Peterson AT, Campbell LP, Soberón J, Ji L, Escobar LE. NicheA: creating virtual species and ecological niches in multivariate environmental scenarios. Ecography. 2016;39(8):805–13.

[39] Boria RA, Olson LE, Goodman SM, Anderson RP. Spatial filtering to reduce sampling bias can improve the performance of ecological niche models. Ecol Model. 2014;275:73–7.

[40] Syfert MM, Smith MJ, Coomes DA. The Effects of Sampling Bias and Model Complexity on the Predictive Performance of MaxEnt Species Distribution Models. PloS one. 2013;8(2):e55158 10.1371/journal.pone.0055158 23457462 PMC3573023

[41] Doosti S, Yaghoobi-Ershadi MR, Schaffner F, Moosa-Kazemi SH, Akbarzadeh K, Gooya MM, et al Mosquito Surveillance and the First Record of the Invasive Mosquito Species *Aedes* (*Stegomyia*) *albopictus* (Skuse) (Diptera: Culicidae) in Southern Iran. Iran J Public Health. 2016;45(8):1064–73. 27928533 PMC5139964

[42] Benallal KE, Allal-Ikhlef A, Benhamouda K, Schaffner F, Harrat Z. First report of *Aedes* (Stegomyia) *albopictus* (Diptera: Culicidae) in Oran, West of Algeria. Acta Trop. 2016;164:411–3. 10.1016/j.actatropica.2016.09.027 27697483

[43] Medlock JM, Vaux AGC, Cull B, Schaffner F, Gillingham E, Pfluger V, et al Detection of the invasive mosquito species *Aedes albopictus* in southern England. Lancet Infect Dis. 2017;17(2):140 10.1016/S1473-3099(17)30024-5 28134111

[44] Abozeid S, Elsayed AK, Schaffner F, Samy AM. Re-emergence of *Aedes aegypti* in Egypt. Lancet Infect Dis. 2018;18(2):142–3. 10.1016/S1473-3099(18)30018-5 29412959

[45] European Centre for Disease Prevention and Control (ECDC). Communicable disease threats report. 2017; Week 51.

[46] Giordano BV, Gasparotto A, Hunter FF. A Checklist of the 67 Mosquito Species of Ontario, Canada. J Am Mosq Control Assoc. 2015;31(1):101–3. 10.2987/14-6456R.1 25843183

[47] Zayed A, Awash AA, Esmail MA, Al-Mohamadi HA, Al-Salwai M, Al-Jasari A, et al Detection of Chikungunya virus in *Aedes aegypti* during 2011 outbreak in Al Hodayda, Yemen. Acta Trop. 2012;123(1):62–6. 10.1016/j.actatropica.2012.03.004 22469818

[48] Derraik JG. Exotic mosquitoes in New Zealand: a review of species intercepted, their pathways and ports of entry. Aust N Z J Public Health. 2004;28(5):433–44. 15707185 10.1111/j.1467-842x.2004.tb00025.x

[49] Brady OJ, Golding N, Pigott DM, Kraemer MU, Messina JP, Reiner RC Jr., et al Global temperature constraints on *Aedes aegypti* and *Ae. albopictus* persistence and competence for dengue virus transmission. Parasit Vectors. 2014;7:338 10.1186/1756-3305-7-338 25052008 PMC4148136

[50] Johnson TL, Haque U, Monaghan AJ, Eisen L, Hahn MB, Hayden MH, et al Modeling the Environmental Suitability for *Aedes* (*Stegomyia*) *aegypti* and *Aedes* (*Stegomyia*) *albopictus* (Diptera: Culicidae) in the Contiguous United States. J Med Entomol. 2017;54(6):1605–14. 10.1093/jme/tjx163 29029153 PMC5868335

[51] Lozano-Fuentes S, Hayden MH, Welsh-Rodriguez C, Ochoa-Martinez C, Tapia-Santos B, Kobylinski KC, et al The dengue virus mosquito vector *Aedes aegypti* at high elevation in Mexico. Am J Trop Med Hyg. 2012;87(5):902–9. 10.4269/ajtmh.2012.12-0244 22987656 PMC3516267

[52] Duncombe J, Espino F, Marollano K, Velazco A, Ritchie SA, Hu WB, et al Characterising the spatial dynamics of sympatric *Aedes aegypti* and *Aedes albopictus* populations in the Philippines. Geospat Health. 2013;8(1):255–65. 10.4081/gh.2013.71 24258900

[53] Minard G, Tran Van V, Tran FH, Melaun C, Klimpel S, Koch LK, et al Identification of sympatric cryptic species of *Aedes albopictus* subgroup in Vietnam: new perspectives in phylosymbiosis of insect vector. Parasit Vectors. 2017;10:276 10.1186/s13071-017-2202-9 28577575 PMC5457575

[54] Braks MAH, Honório NA, Lounibos LP, Lourenço-De-Oliveira R, Juliano SA. Interspecific Competition Between Two Invasive Species of Container Mosquitoes, *Aedes aegypti* and *Aedes albopictus* (Diptera: Culicidae), in Brazil. Ann Entomol Soc Am. 2004;97(1):130–9.

[55] Thomas SM, Obermayr U, Fischer D, Kreyling J, Beierkuhnlein C. Low-temperature threshold for egg survival of a post-diapause and non-diapause European aedine strain, *Aedes albopictus* (Diptera: Culicidae). Parasites & vectors. 2012;5:100. 22621367 10.1186/1756-3305-5-100 PMC3403971

[56] Bargielowski IE, Lounibos LP, Carrasquilla MC. Evolution of resistance to satyrization through reproductive character displacement in populations of invasive dengue vectors. Proc Natl Acad Sci U S A. 2013;110(8):2888–92. 10.1073/pnas.1219599110 23359710 PMC3581888

[57] Obenauer JF, Andrew Joyner T, Harris JB. The importance of human population characteristics in modeling *Aedes aegypti* distributions and assessing risk of mosquito-borne infectious diseases. Trop Med Health. 2017;45:38 10.1186/s41182-017-0078-1 29167627 PMC5688614

