# Supplementary material for: Mapping the global potential distributions of two arboviral vectors Aedes aegypti and Ae. albopictus under changing climate
Source: PLoS One. 2018 Dec 31;13(12):e0210122. doi: 10.1371/journal.pone.0210122 (PMC6312308; doi:10.1371/journal.pone.0210122)
Supplement: S1 File — (PDF) [file pone.0210122.s001.pdf]

S1 File: List of general circulation models used in the ecological niche modeling analyses of *Aedes aegypti* and *Ae. albopictus* under future climatic conditions.

**Name of GCM***	**Code**
**BCC-CSM1-1**	**BC**
**CCSM4**	**CC**
**GISS-E2-R**	**GS**
**HadGEM2-AO**	**HD**
**HadGEM2-ES**	**HE**
**IPSL-CM5A-LR**	**IP**
**MIROC-ESM-CHEM**	**MI**
**MIROC5**	**MC**
**MRI-CGCM3**	**MG**

* We removed two additional common GCMs (NorESM 1-M, and MIROC-ESM) due to problems in some of their variables. So, the analyses used only 9 GCMs/RCP/Year.
