# Supplementary material for: Mapping the global potential distributions of two arboviral vectors Aedes aegypti and Ae. albopictus under changing climate
Source: PLoS One. 2018 Dec 31;13(12):e0210122. doi: 10.1371/journal.pone.0210122 (PMC6312308; doi:10.1371/journal.pone.0210122)
Supplement: S2 File — Navy blue shaded areas were modeled as suitable; gray areas were modeled as unsuitable. (PDF) [file pone.0210122.s002.pdf]

S2 File. Current potential distribution of *Aedes aegypti* in Spanish islands based on present-day conditions. Navy blue shaded areas were modeled as suitable; gray areas were modeled as unsuitable.

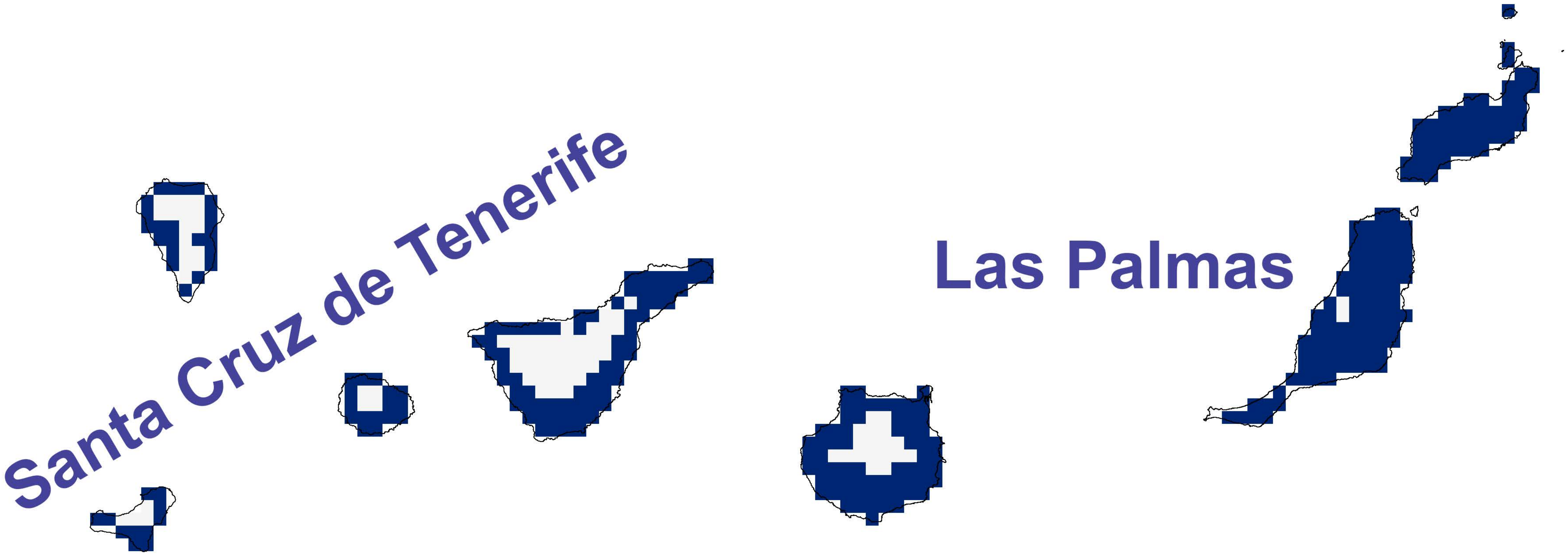
Santa Cruz de Tenerife
Las Palmas
