# Supplementary material for: Mapping the global potential distributions of two arboviral vectors Aedes aegypti and Ae. albopictus under changing climate
Source: PLoS One. 2018 Dec 31;13(12):e0210122. doi: 10.1371/journal.pone.0210122 (PMC6312308; doi:10.1371/journal.pone.0210122)
Supplement: S3 File — Navy blue shaded areas were modeled as suitable; gray areas were modeled as unsuitable. (PDF) [file pone.0210122.s003.pdf]

S3 File. Current potential distribution of *Aedes aegypti* in Sudan, and Red Sea coasts in Egypt and Saudi Arabia based on present-day conditions. Navy blue shaded areas were modeled as suitable; gray areas were modeled as unsuitable.

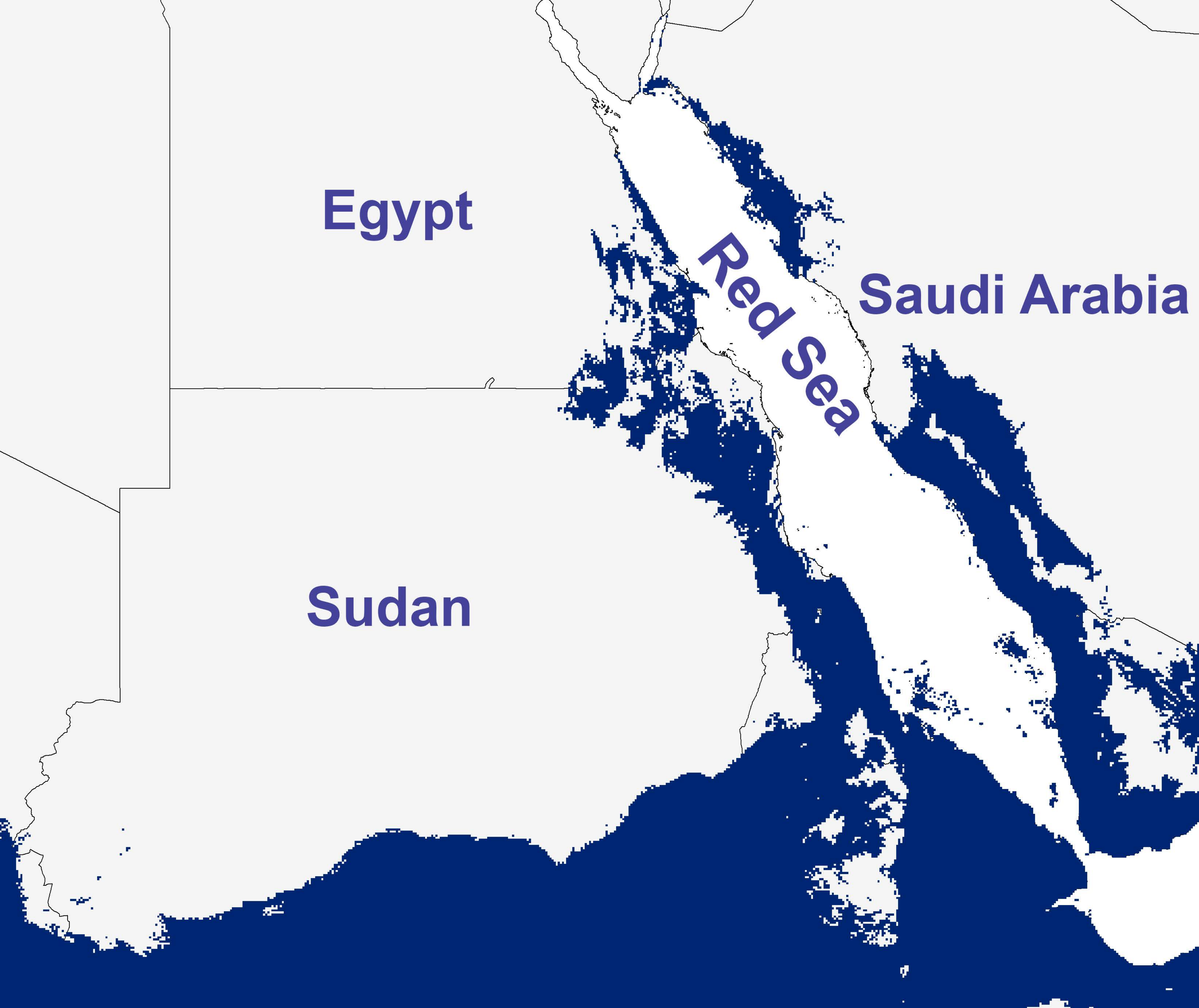
Egypt
Red Sea
Saudi Arabia
Sudan

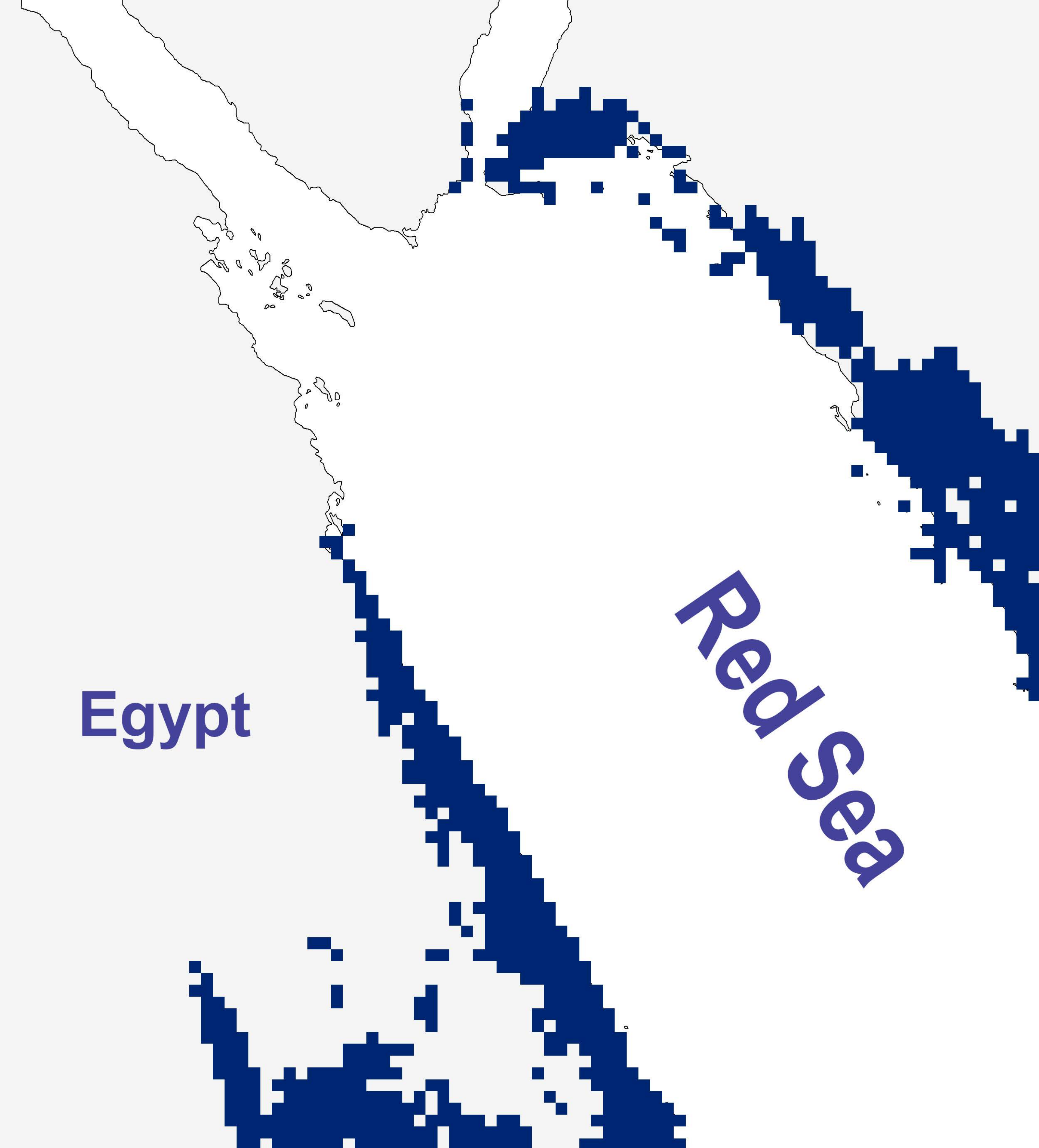

Egypt
Red Sea
