# Supplementary material for: Mapping the global potential distributions of two arboviral vectors Aedes aegypti and Ae. albopictus under changing climate
Source: PLoS One. 2018 Dec 31;13(12):e0210122. doi: 10.1371/journal.pone.0210122 (PMC6312308; doi:10.1371/journal.pone.0210122)
Supplement: S6 File — Yellow points are independent occurrence data from the Old World and North America. Blue areas are represented as suitable and gray as unsuitable. (PDF) [file pone.0210122.s006.pdf]

S6 File. Relationship of additional independent records of *Ae. aegypti* and *Ae. albopictus* to areas predicted as suitable for *Ae. aegypti* and *Ae. albopictus*, respectively. Yellow points are independent occurrence data from the Old World and North America. Blue areas are represented as suitable and gray as unsuitable.

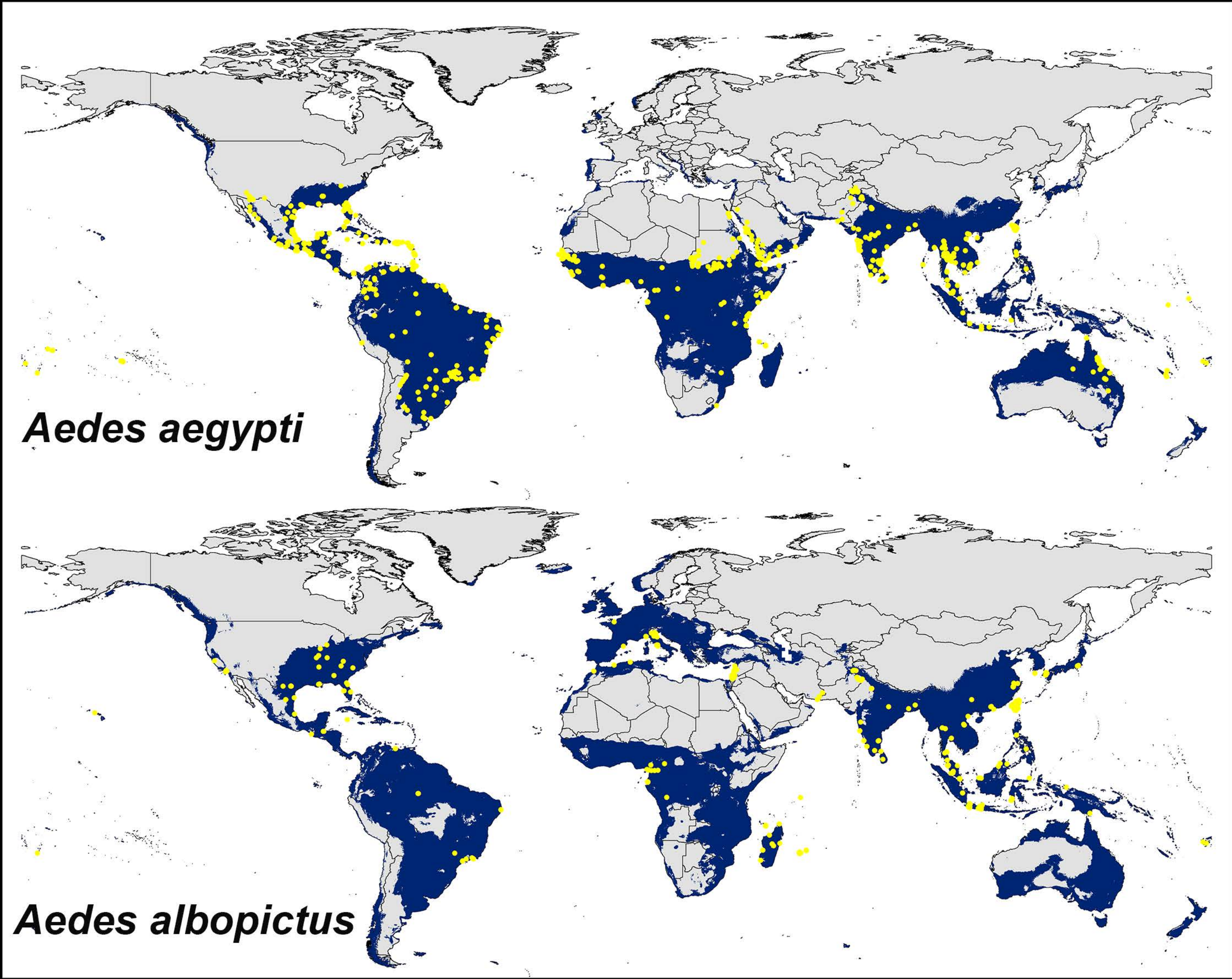
Aedes aegypti
Aedes albopictus
