# Supplementary material for: Mapping the global potential distributions of two arboviral vectors Aedes aegypti and Ae. albopictus under changing climate
Source: PLoS One. 2018 Dec 31;13(12):e0210122. doi: 10.1371/journal.pone.0210122 (PMC6312308; doi:10.1371/journal.pone.0210122)
Supplement: S8 File — Brown areas are modeled suitable conditions; gray areas are unsuitable conditions. (PDF) [file pone.0210122.s008.pdf]

S8 File. Predicted future potential distribution of *Aedes albopictus* under four future representative concentration pathways of climate conditions in 2070. Brown areas are modeled suitable conditions; gray areas are unsuitable conditions.

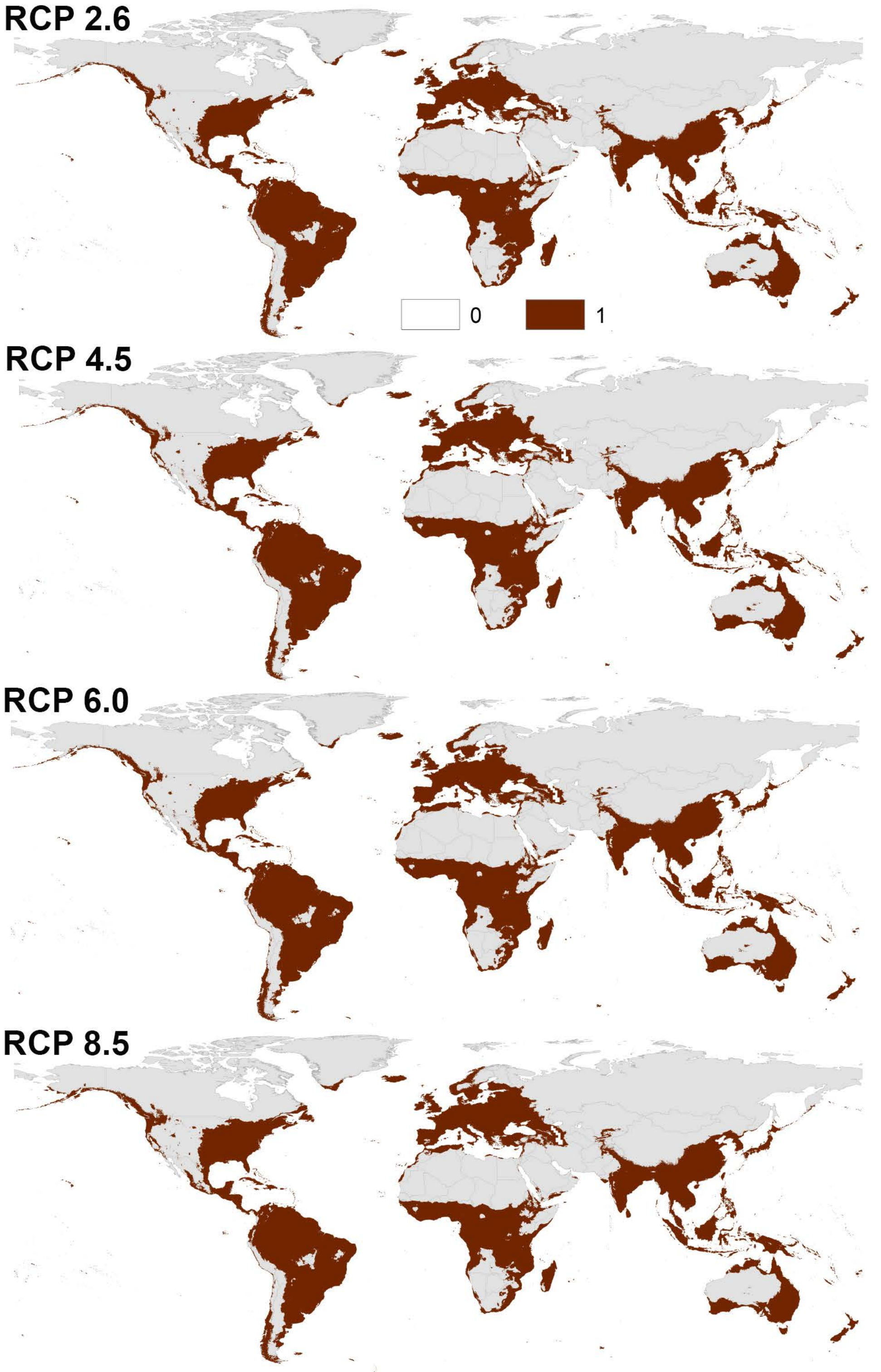
RCP 2.6
0
1
RCP 4.5
RCP 6.0
RCP 8.5
