# Supplementary material for: Mapping the global potential distributions of two arboviral vectors Aedes aegypti and Ae. albopictus under changing climate
Source: PLoS One. 2018 Dec 31;13(12):e0210122. doi: 10.1371/journal.pone.0210122 (PMC6312308; doi:10.1371/journal.pone.0210122)
Supplement: S9 File — Navy blue represents model stability under both current and future conditions, dark orange represents agreement among all climate models in anticipating the potential distributional areas in the future, and light orange indicates low agreement between diverse models as regards distributional potential in the future. (PDF) [file pone.0210122.s009.pdf]

S9 File. Summary of the modeled global distribution of *Aedes aegypti* under both current and future climatic conditions in 2070 showing stability of predictions at present and into the future, and to illustrate differences among representative concentration pathways (RCPs). Navy blue represents model stability under both current and future conditions, dark orange represents agreement among all climate models in anticipating the potential distributional areas in the future, and light orange indicates low agreement between diverse models as regards distributional potential in the future.

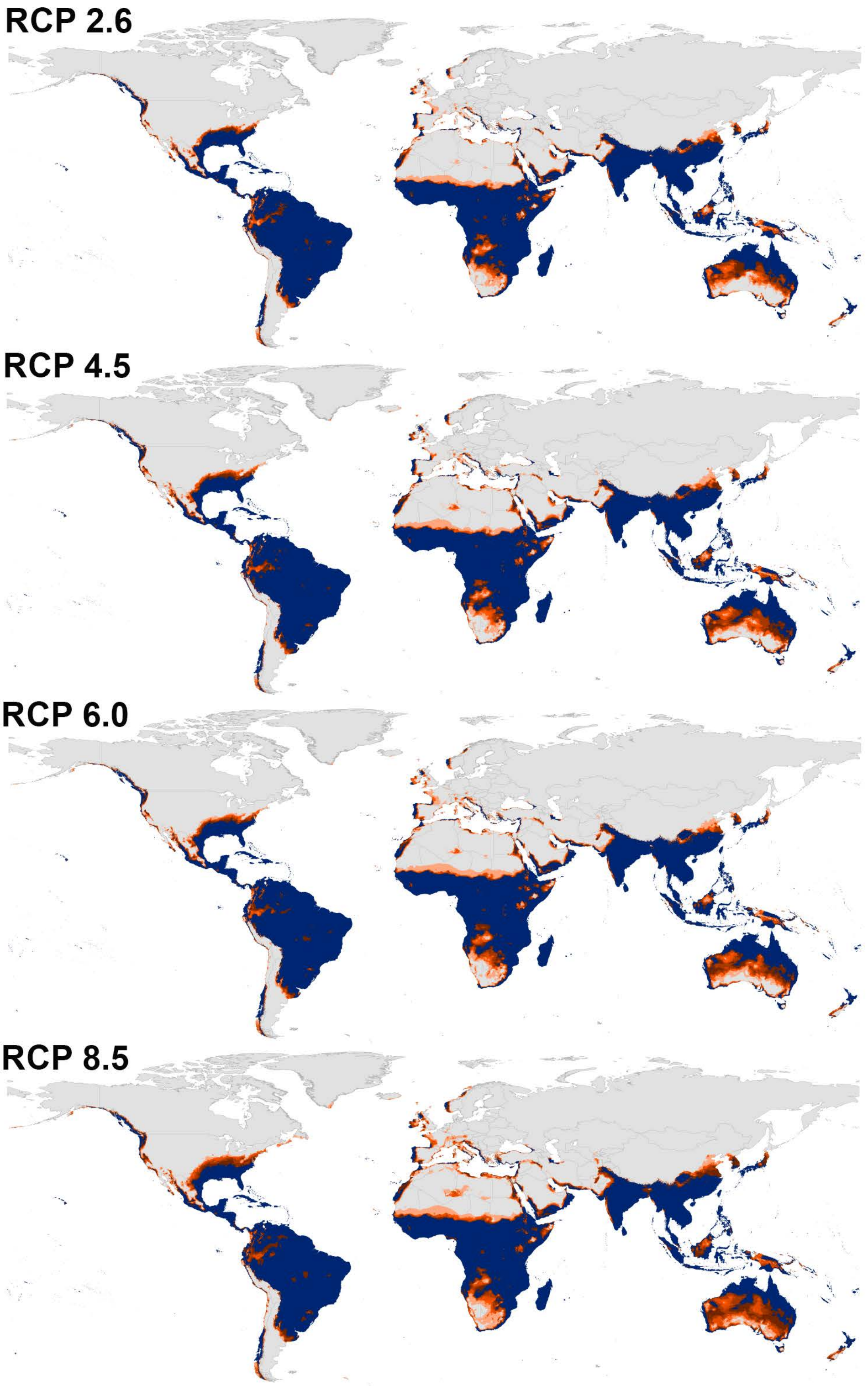
RCP 2.6
RCP 4.5
RCP 6.0
RCP 8.5
