# Supplementary material for: Mapping the global potential distributions of two arboviral vectors Aedes aegypti and Ae. albopictus under changing climate
Source: PLoS One. 2018 Dec 31;13(12):e0210122. doi: 10.1371/journal.pone.0210122 (PMC6312308; doi:10.1371/journal.pone.0210122)
Supplement: S11 File — (PDF) [file pone.0210122.s011.pdf]

S11 File. Visualization of uncertainty estimate associated with present-day and future predictions of *Aedes aegypti* and *Ae. albopictus*.

I. Visualization of uncertainty estimate associated with present-day predictions of *Aedes aegypti*. Uncertainty index was derived from diverse sets of occurrence in MAXENT samples; uncertainty index was estimated from the range (maximum—minimum) of predictions in 10 replicate runs in MAXENT.

A) Global uncertainty map
B) Close-up to South Asia showing the diverse values of uncertainty index.
C) Close-up to Europe and North Africa showing the diverse values of uncertainty index.
D) Close-up to Sub-Saharan Africa showing the diverse values of uncertainty index.
E) Close-up to North America showing the diverse values of uncertainty index.
F) Close-up to South America showing the diverse values of uncertainty index.

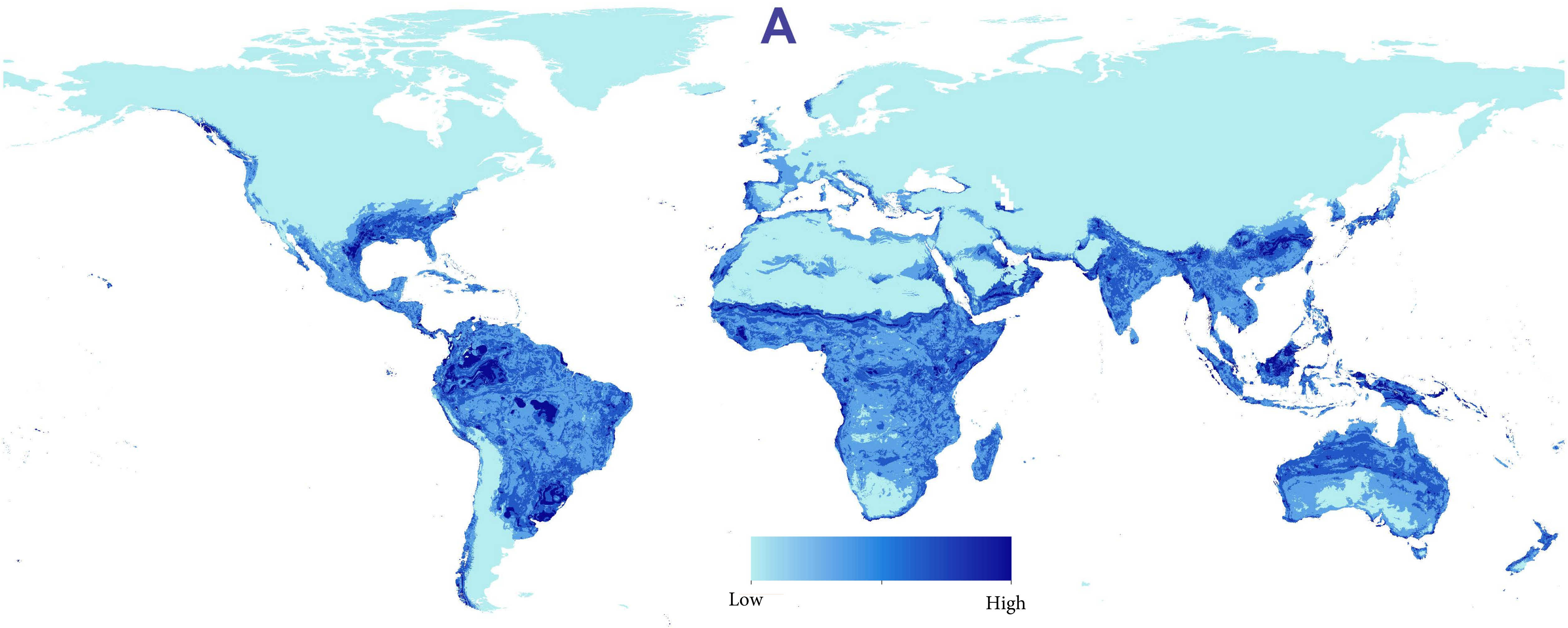

A
Low
High

B

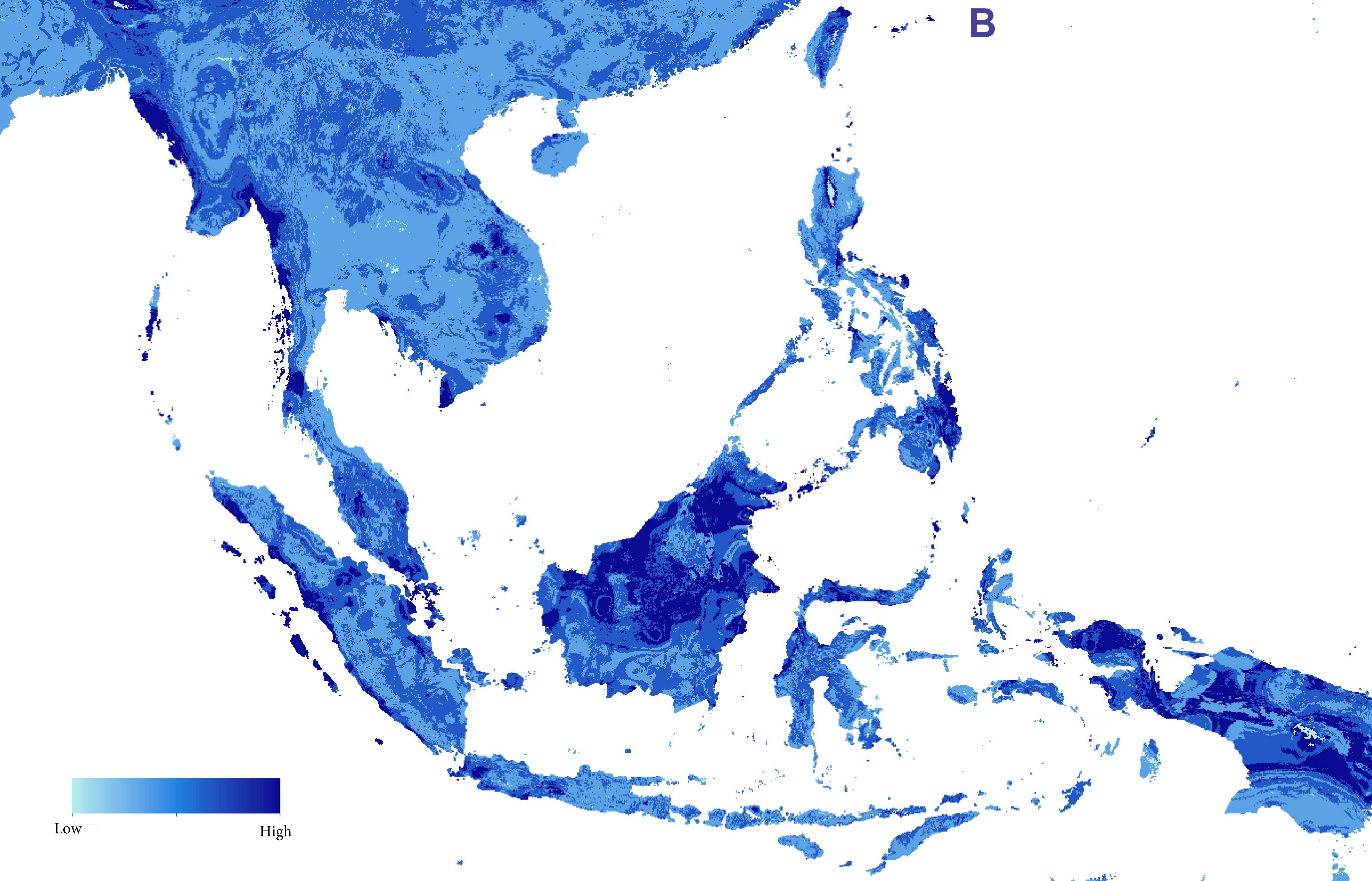

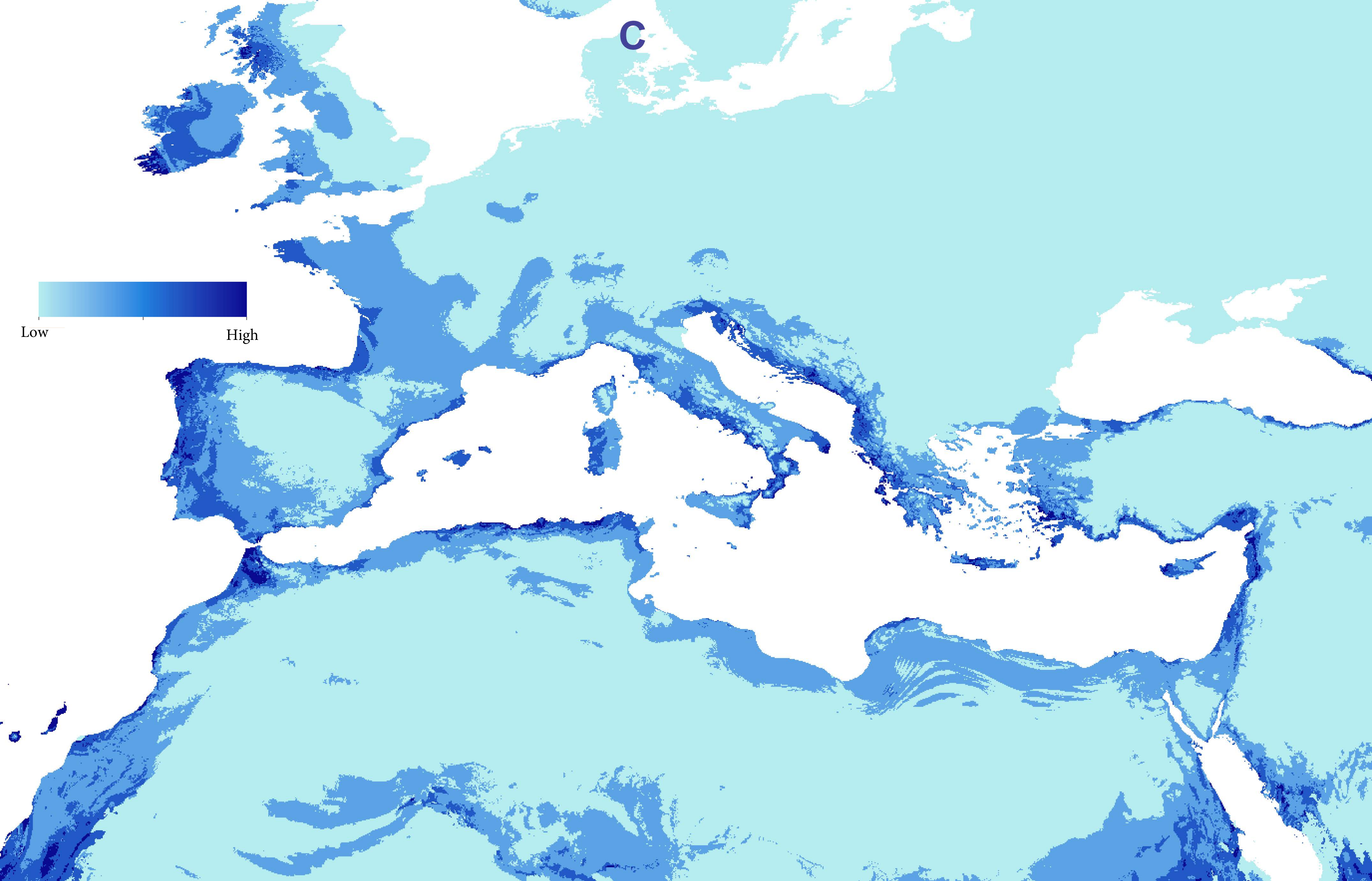
C
Low
High

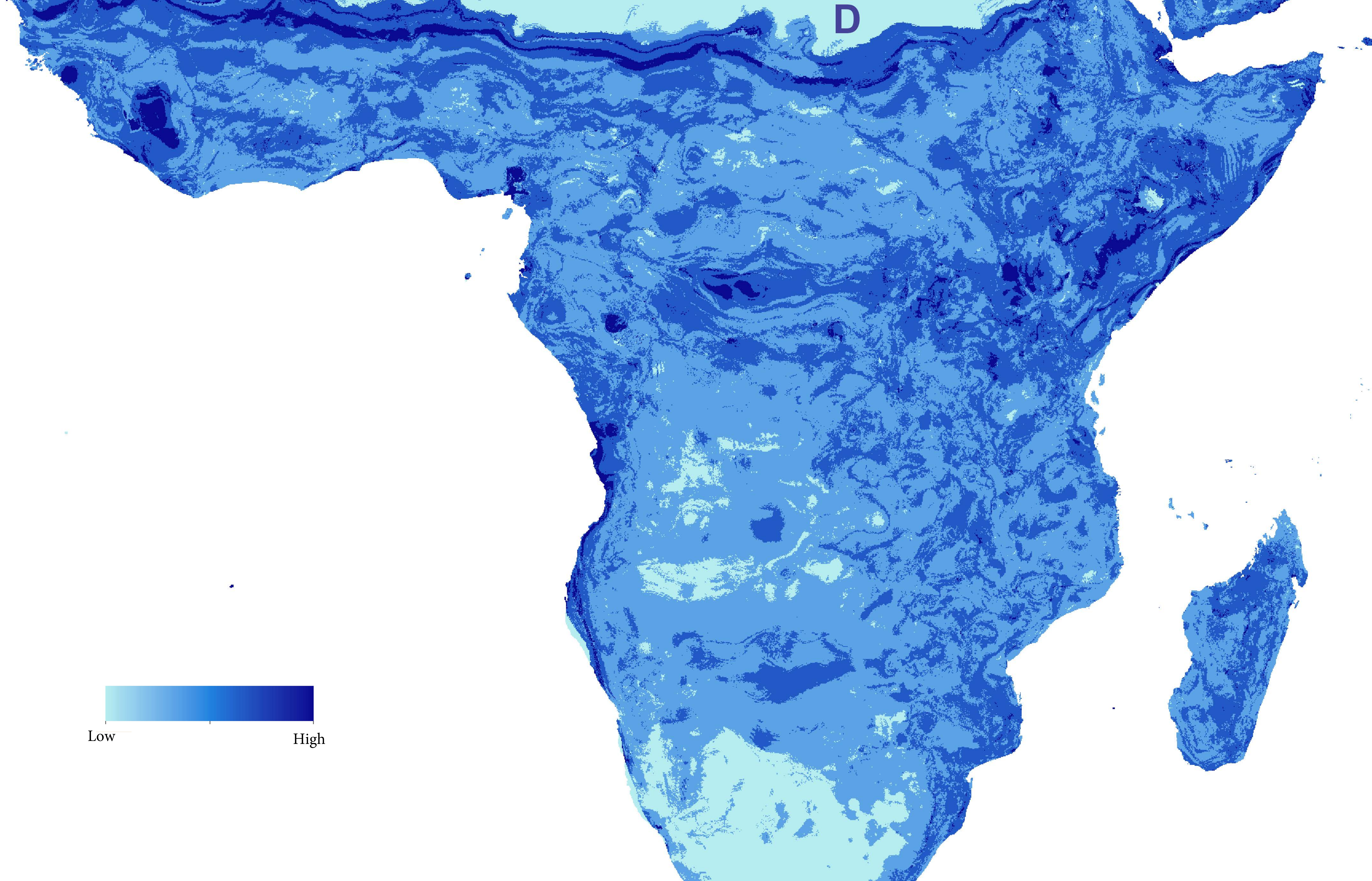
D
Low
High

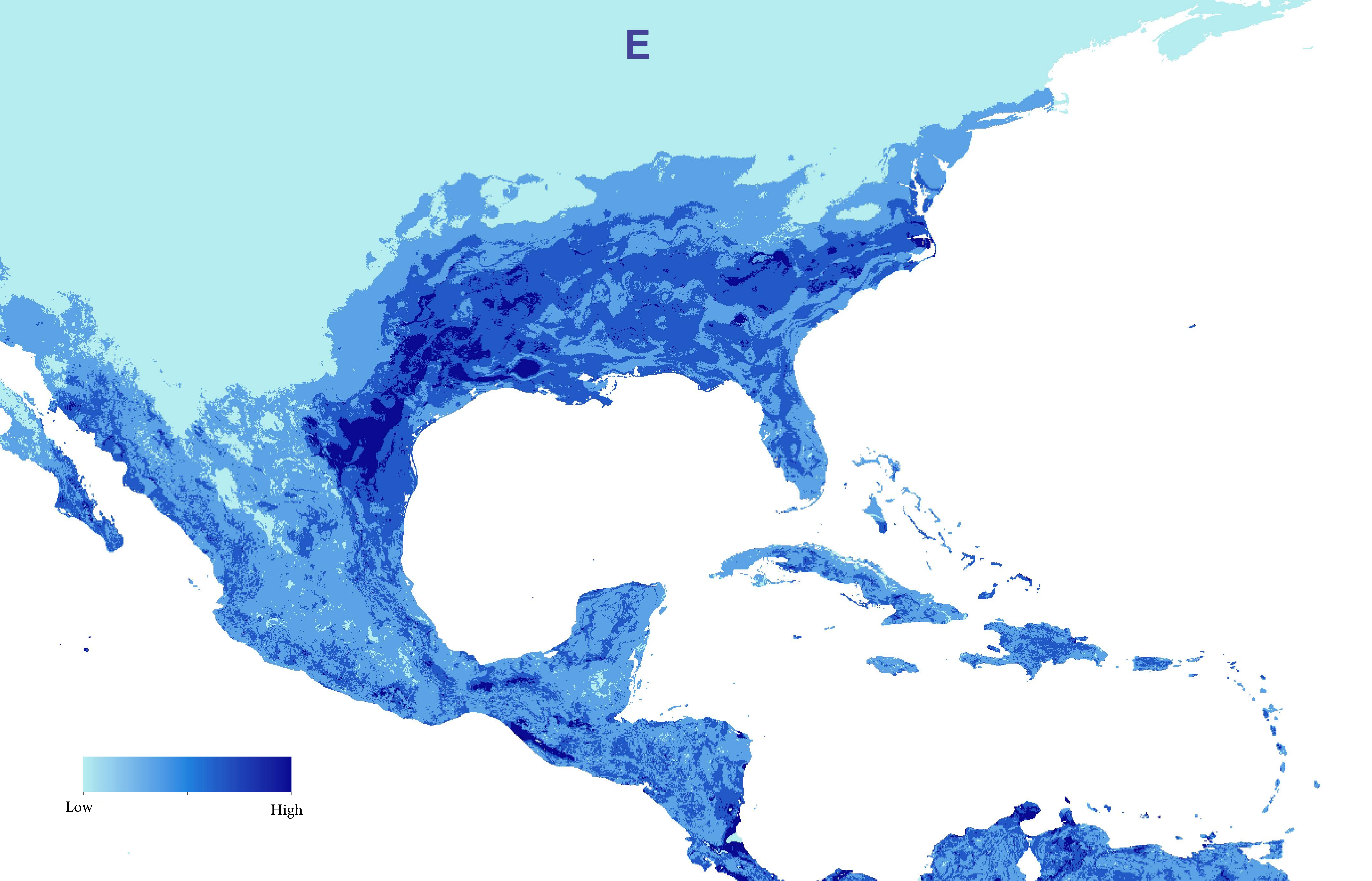

E
Low
High

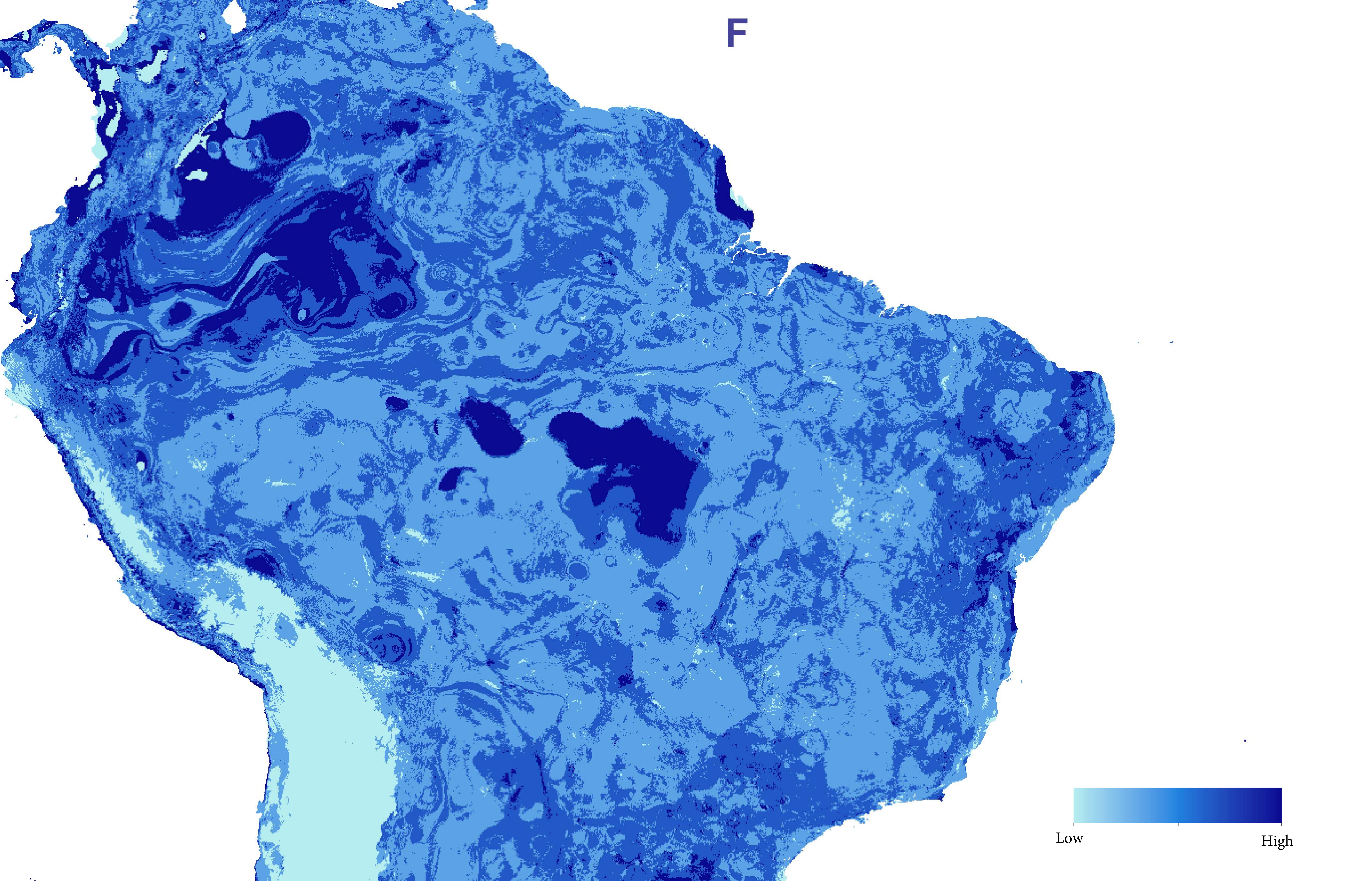
F
Low
High

II. Visualization of uncertainty estimate associated with present-day predictions of *Aedes albopictus*. Uncertainty index was derived from diverse sets of occurrence in MAXENT samples; uncertainty index was estimated from the range (maximum—minimum) of predictions in 10 replicate runs in MAXENT.

A) Global uncertainty map
B) Close-up to South Asia showing the diverse values of uncertainty index.
C) Close-up to Europe and North Africa showing the diverse values of uncertainty index.
D) Close-up to Sub-Saharan Africa showing the diverse values of uncertainty index.
E) Close-up to North America showing the diverse values of uncertainty index.
F) Close-up to South America showing the diverse values of uncertainty index.

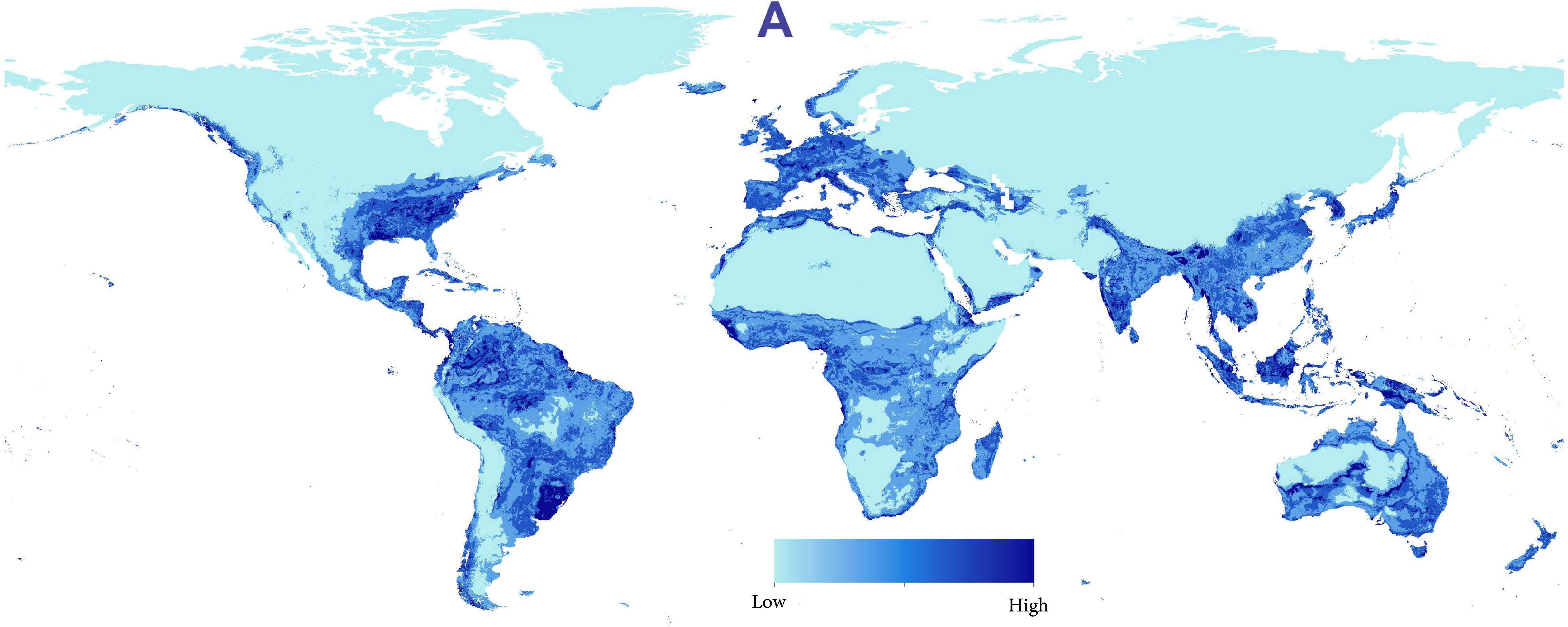
A
Low
High

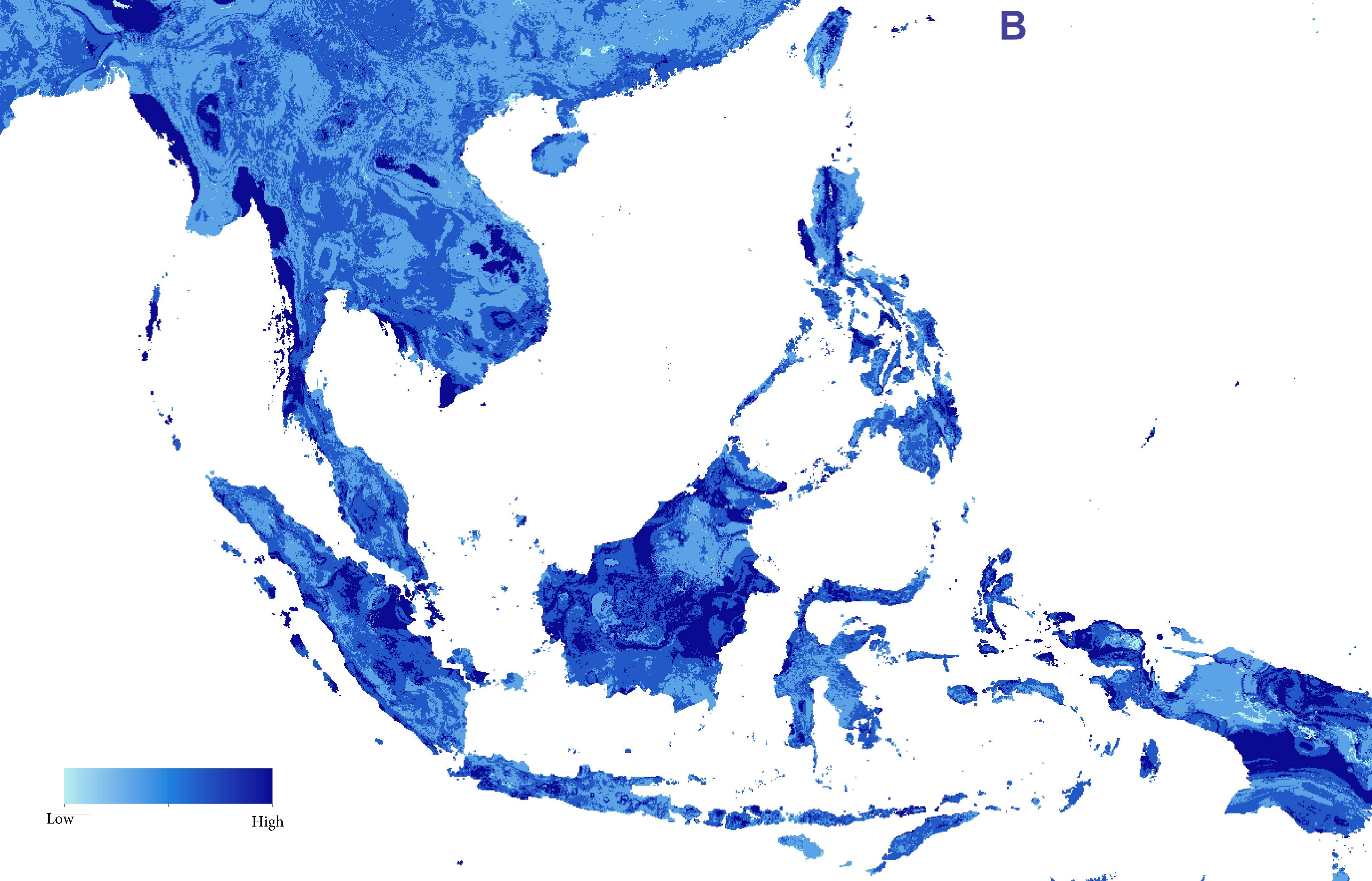
B
Low
High

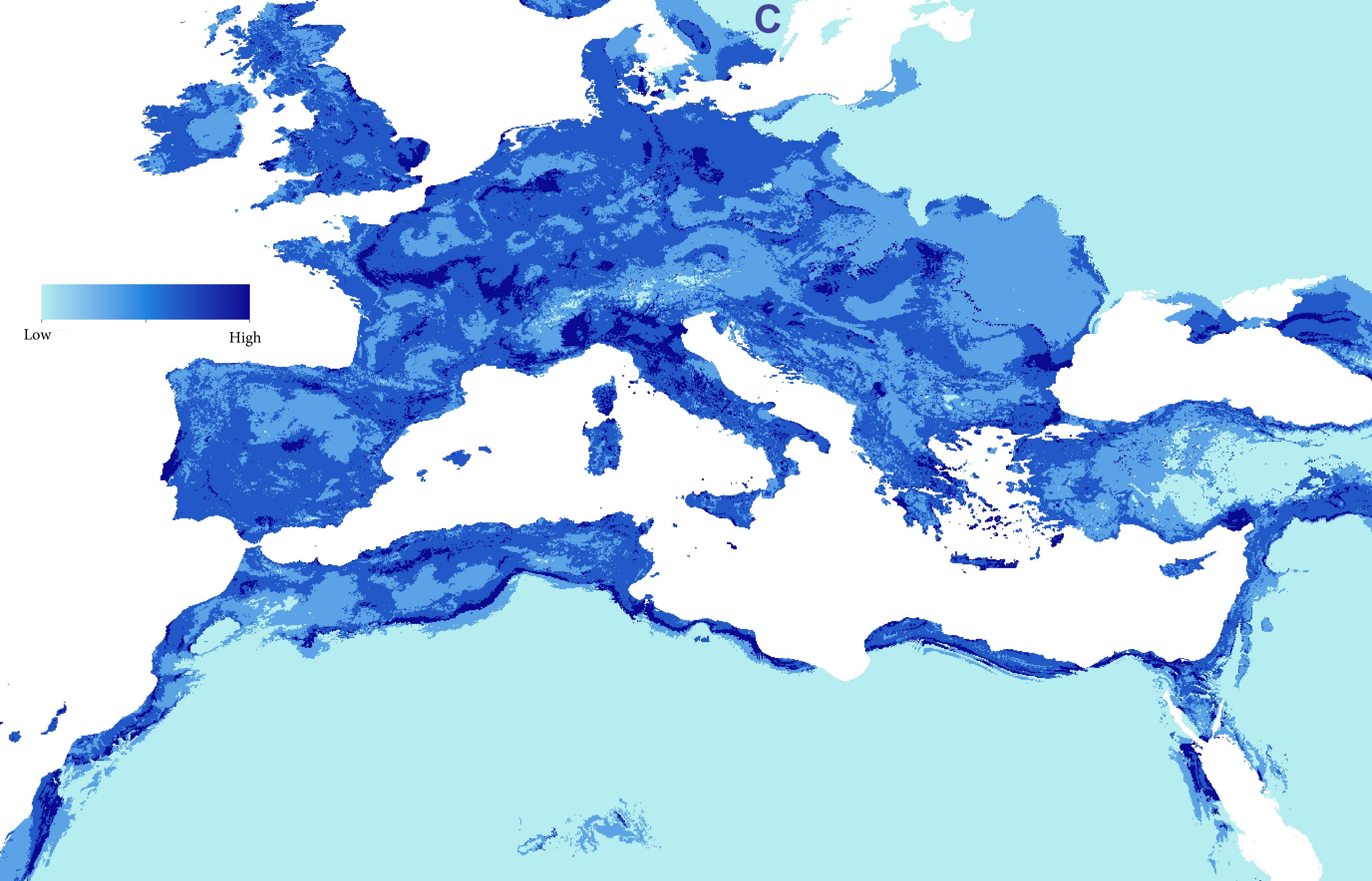
C
Low
High

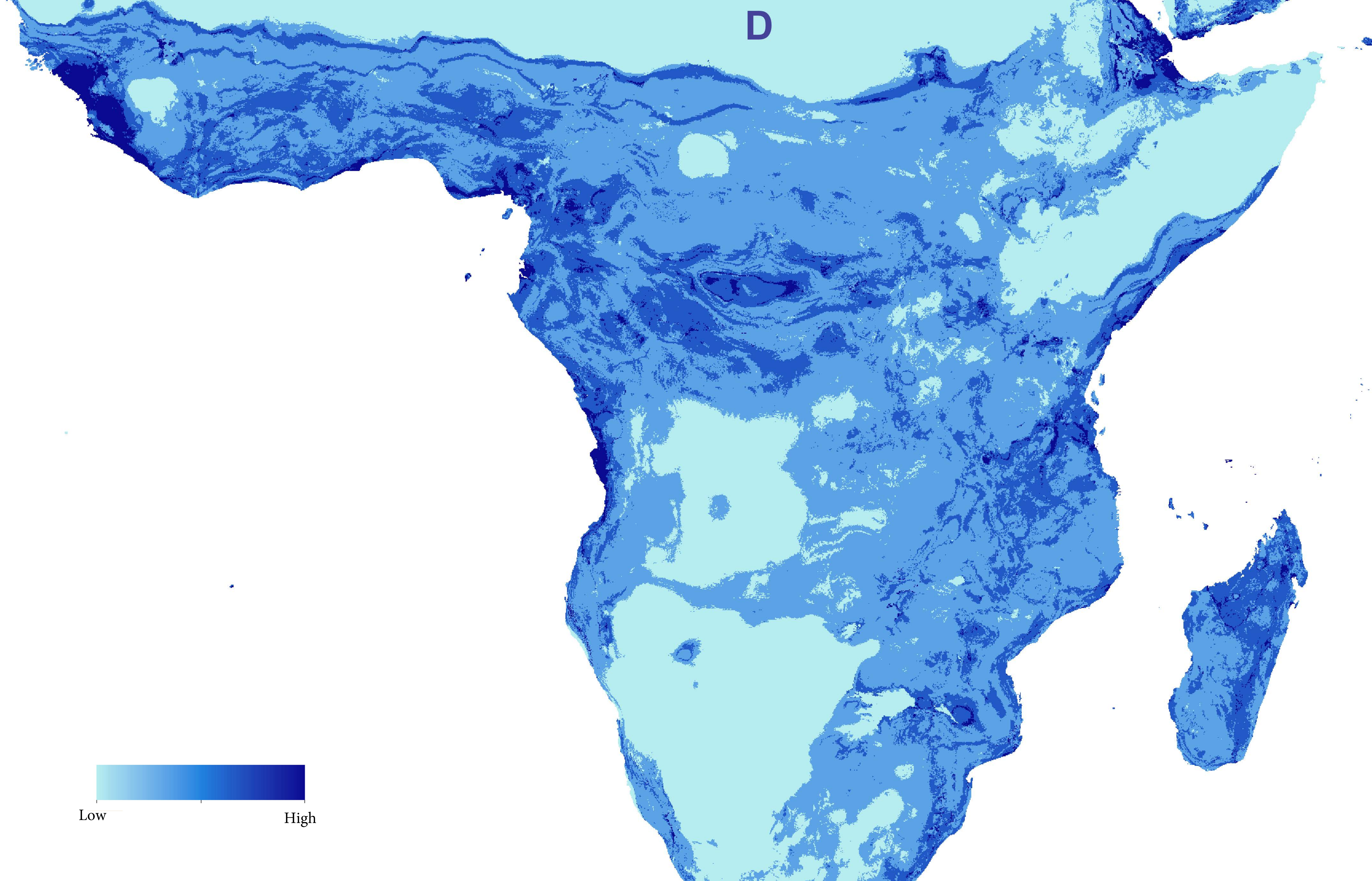
D
Low
High

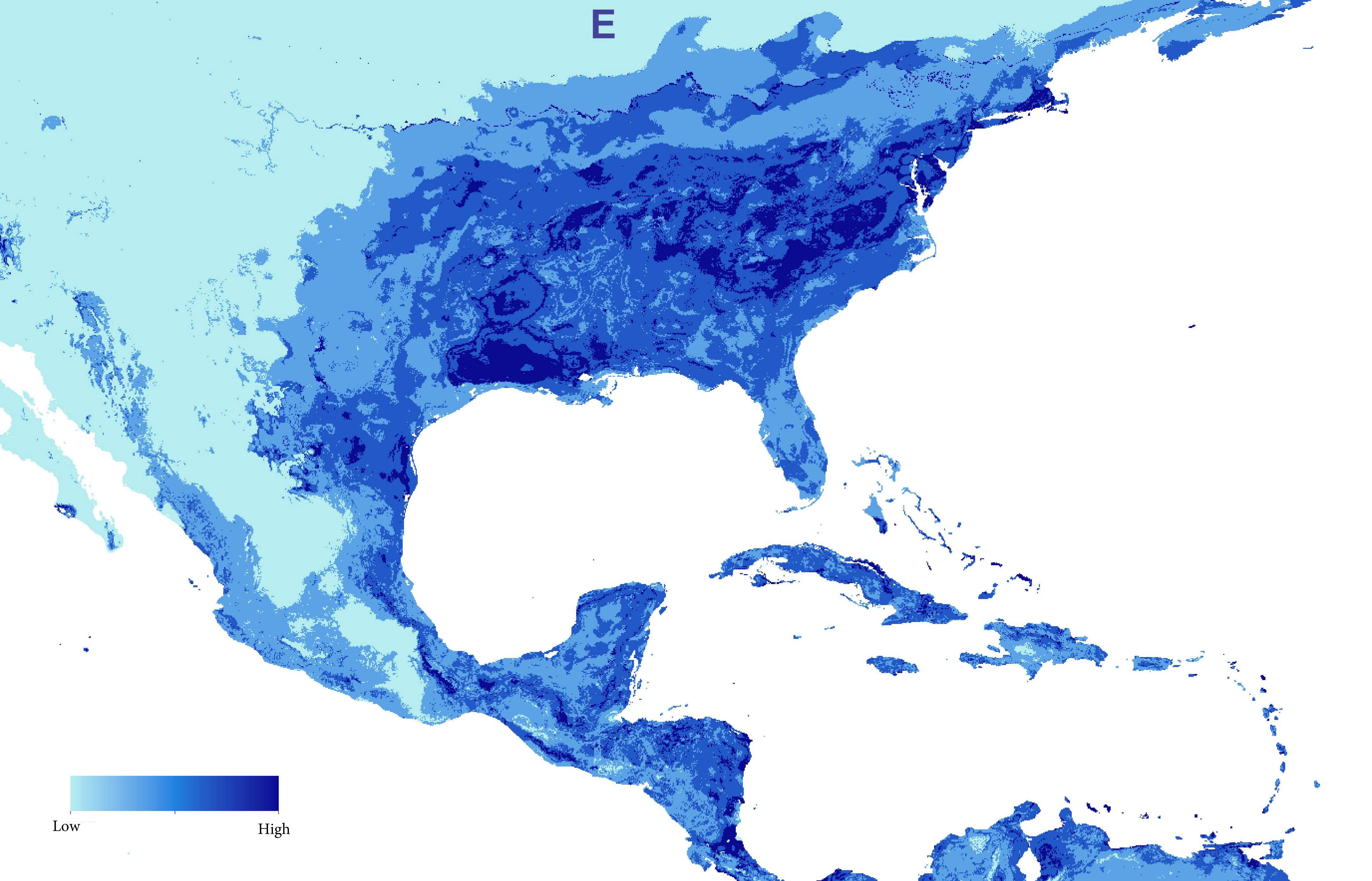
E
Low
High

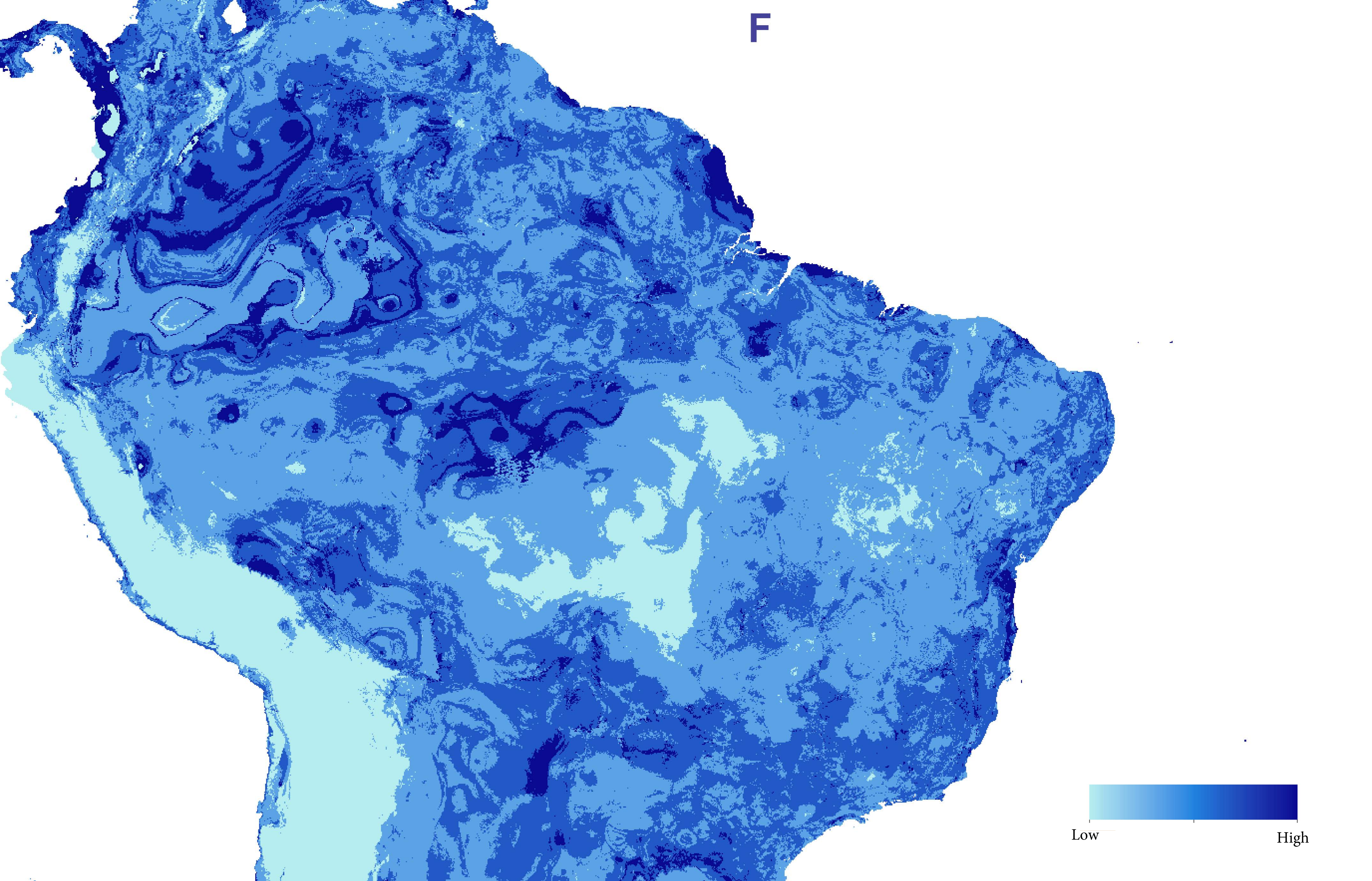
F
Low
High

III. Visualization of uncertainty estimate associated with future predictions of *Aedes aegypti* based on diverse representative concentration pathways (RCPs) in 2050. Uncertainty index was derived from diverse GCMs in each RCP in the study; uncertainty index was estimated as the range (maximum —minimum) across all combinations of GCMs in each RCP.

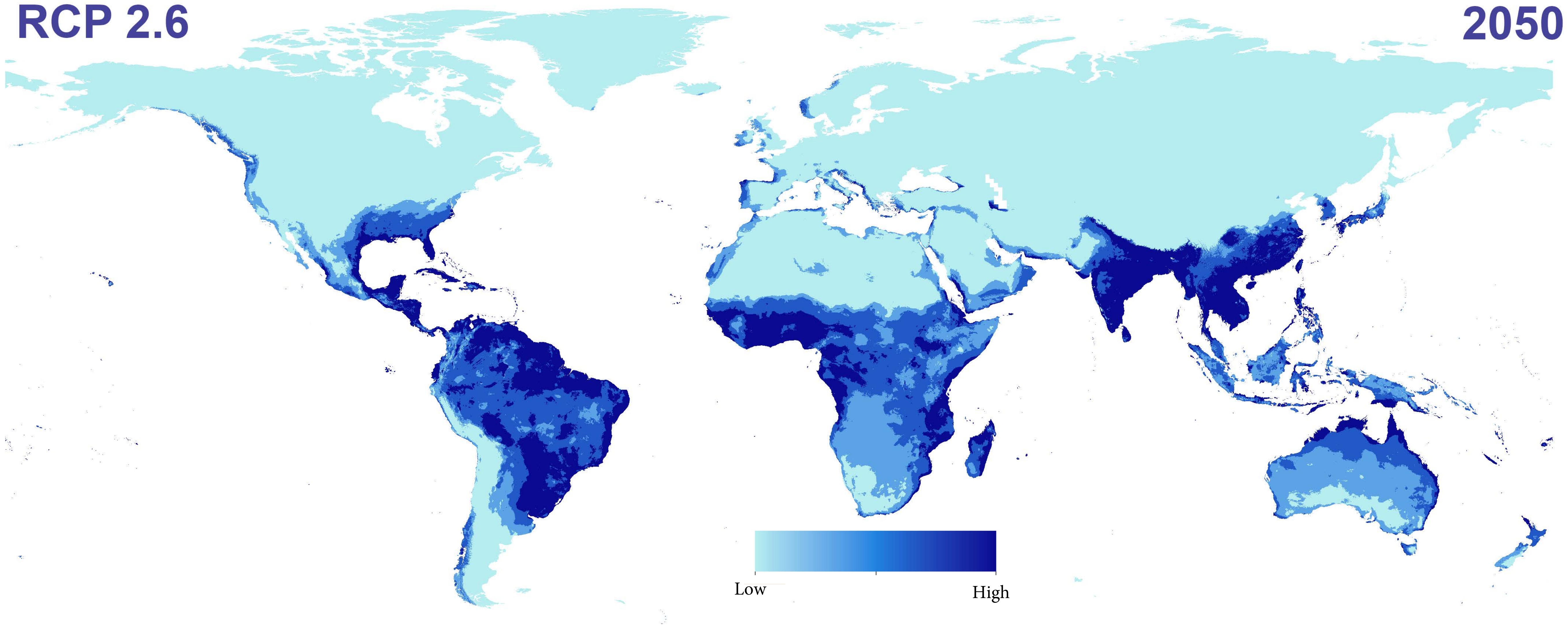
RCP 2.6
2050
Low
High

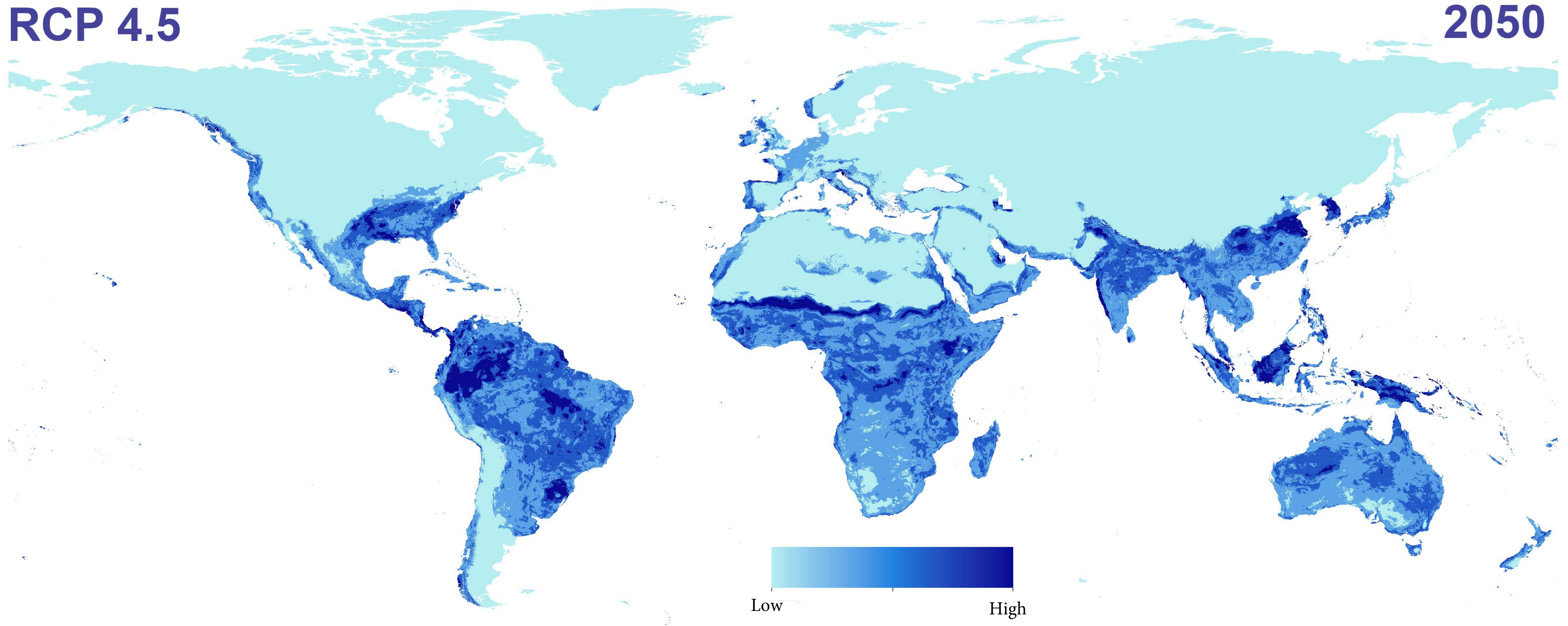

RCP 4.5
2050
Low
High

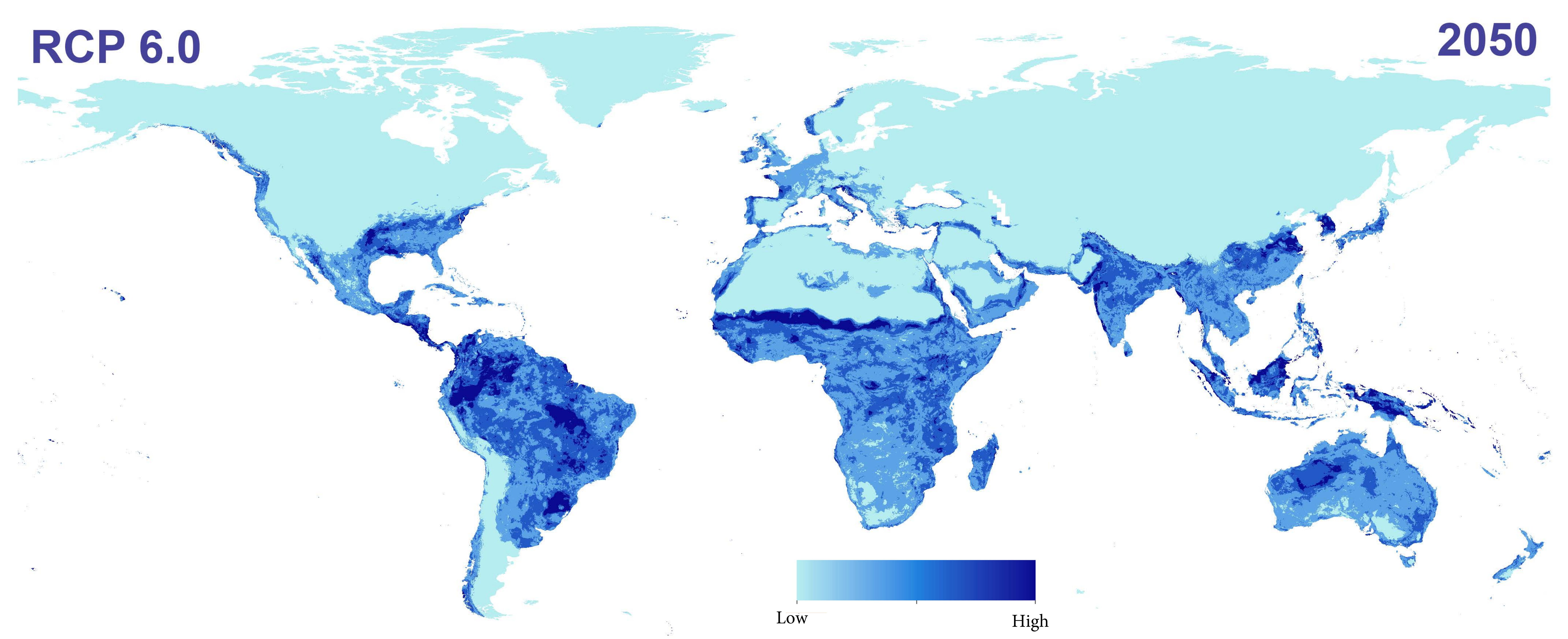
RCP 6.0
2050
Low
High

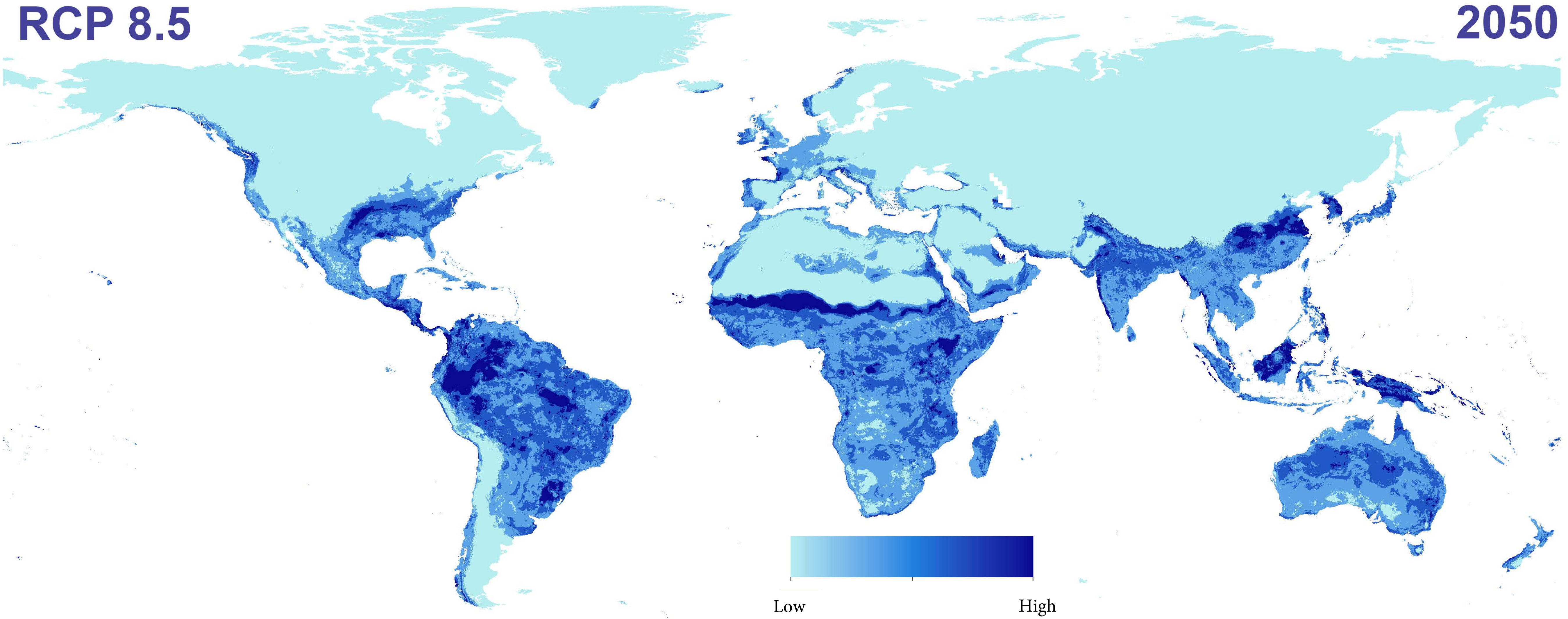
RCP 8.5
2050
Low
High

IV. Visualization of uncertainty estimate associated with future predictions of *Aedes aegypti* based on diverse representative concentration pathways (RCPs) in 2070. Uncertainty index was derived from diverse GCMs in each RCP in the study; uncertainty index was estimated as the range (maximum —minimum) across all combinations of GCMs in each RCP.

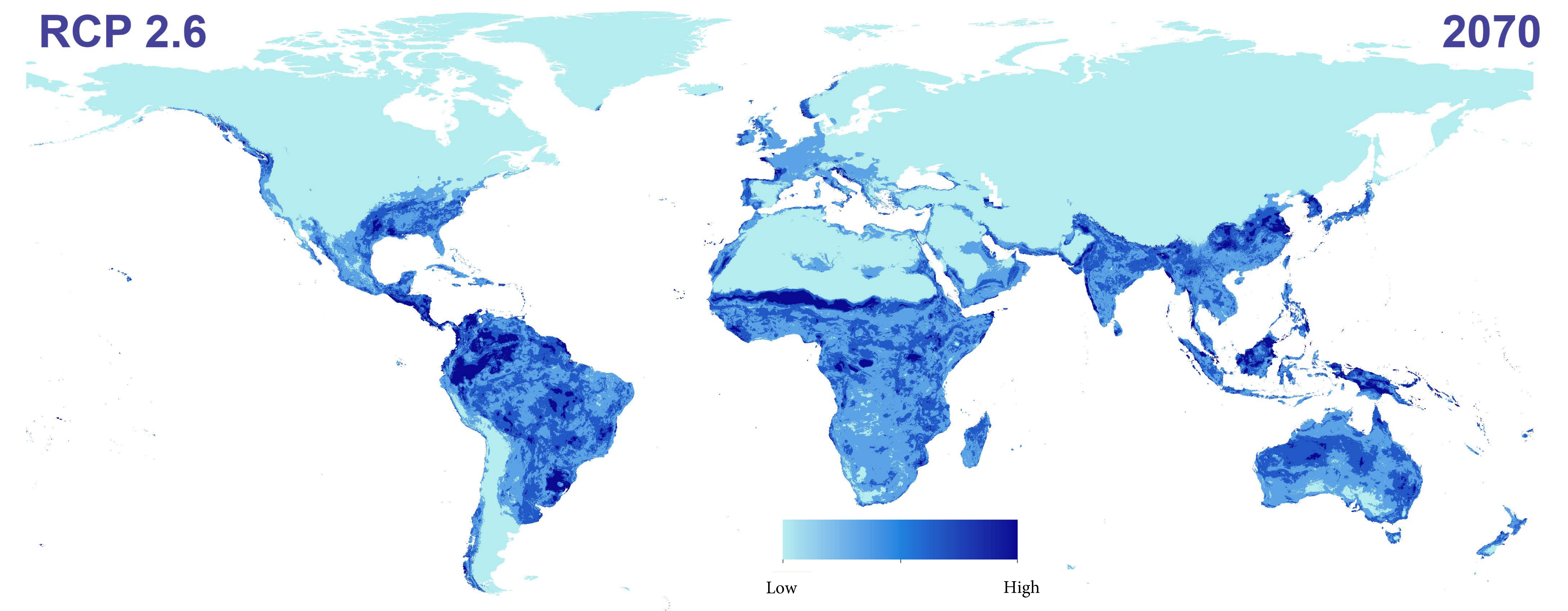
RCP 2.6
2070
Low
High

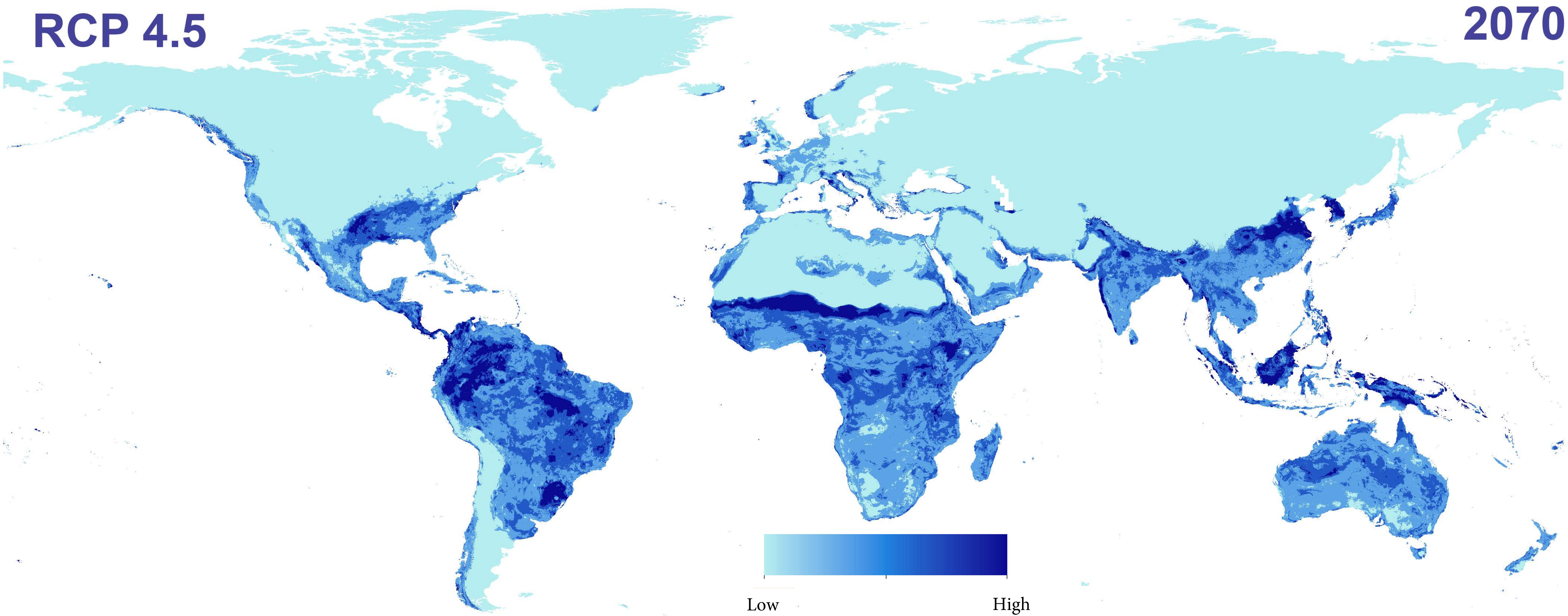
RCP 4.5
2070
Low
High

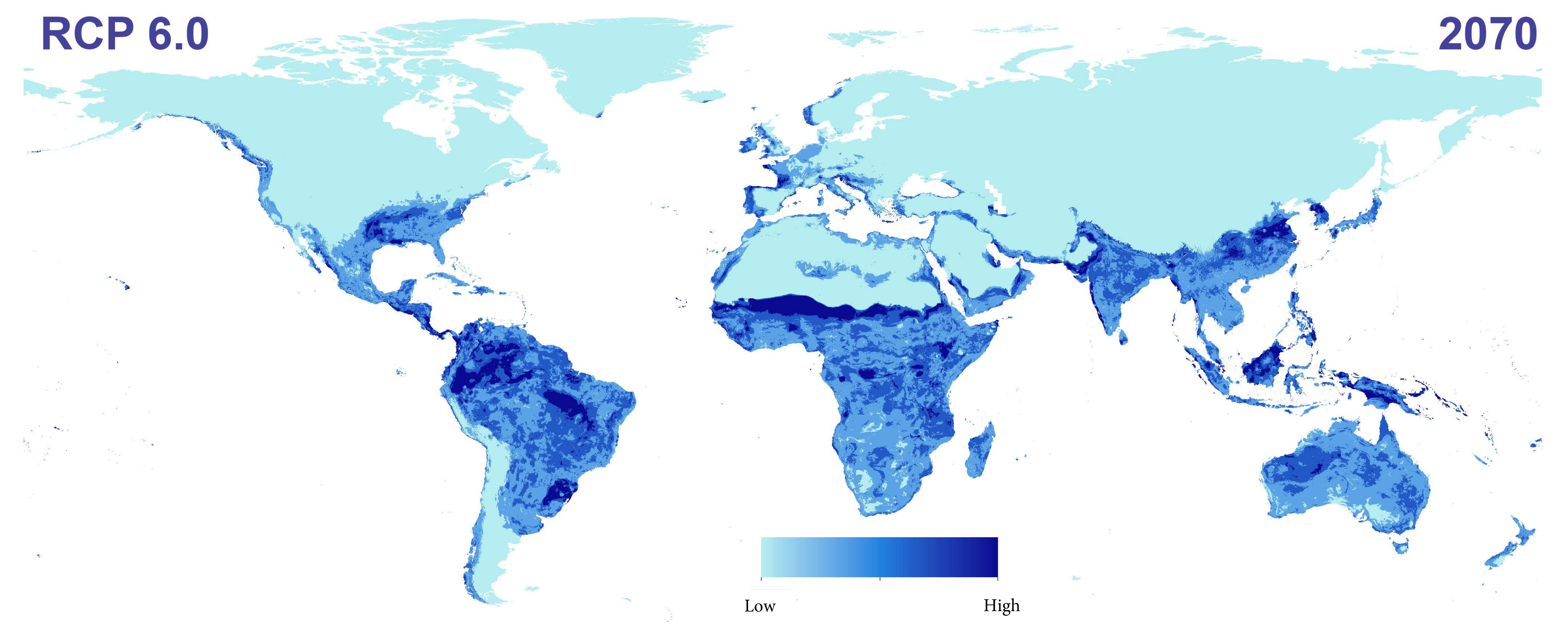
RCP 6.0
2070
Low
High

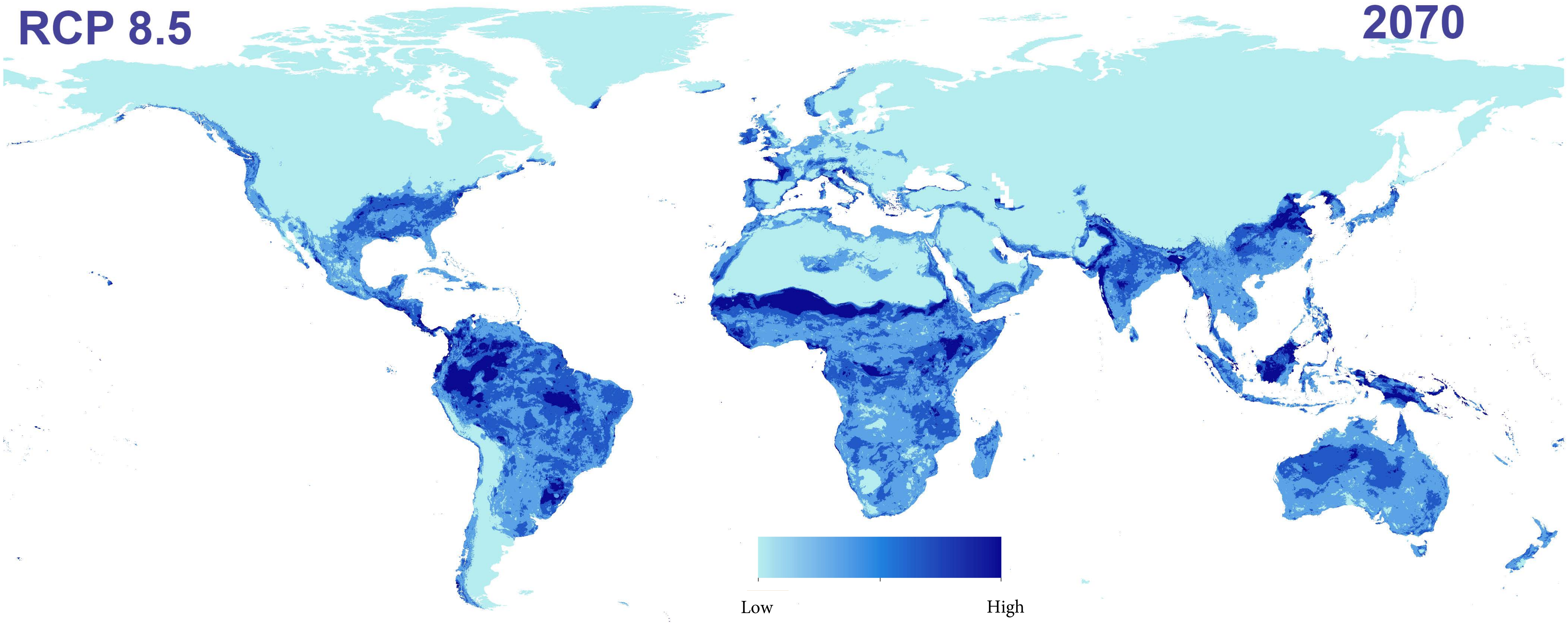
RCP 8.5
2070
Low
High

V. Visualization of uncertainty estimate associated with future predictions of *Aedes albopictus* based on diverse representative concentration pathways (RCPs) in 2050. Uncertainty index was derived from diverse GCMs in each RCP in the study; uncertainty index was estimated as the range (maximum—minimum) across all combinations of GCMs in each RCP.

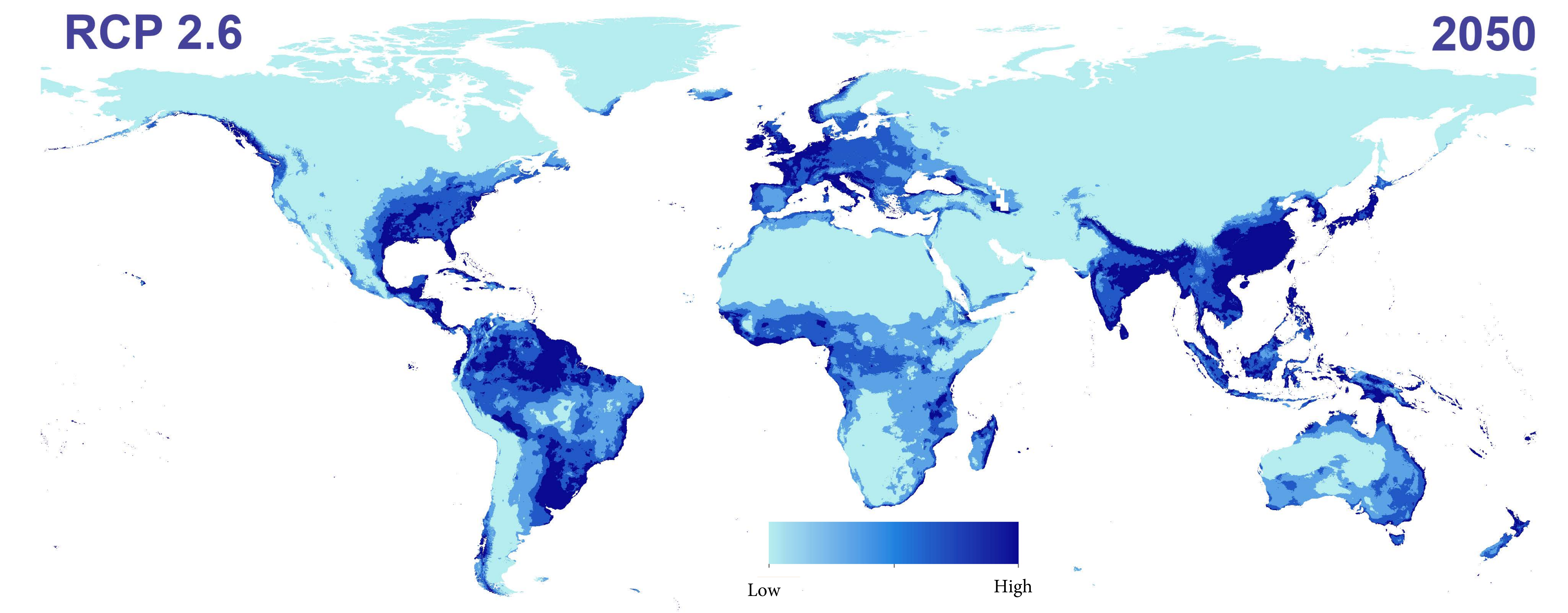
RCP 2.6
2050
Low
High

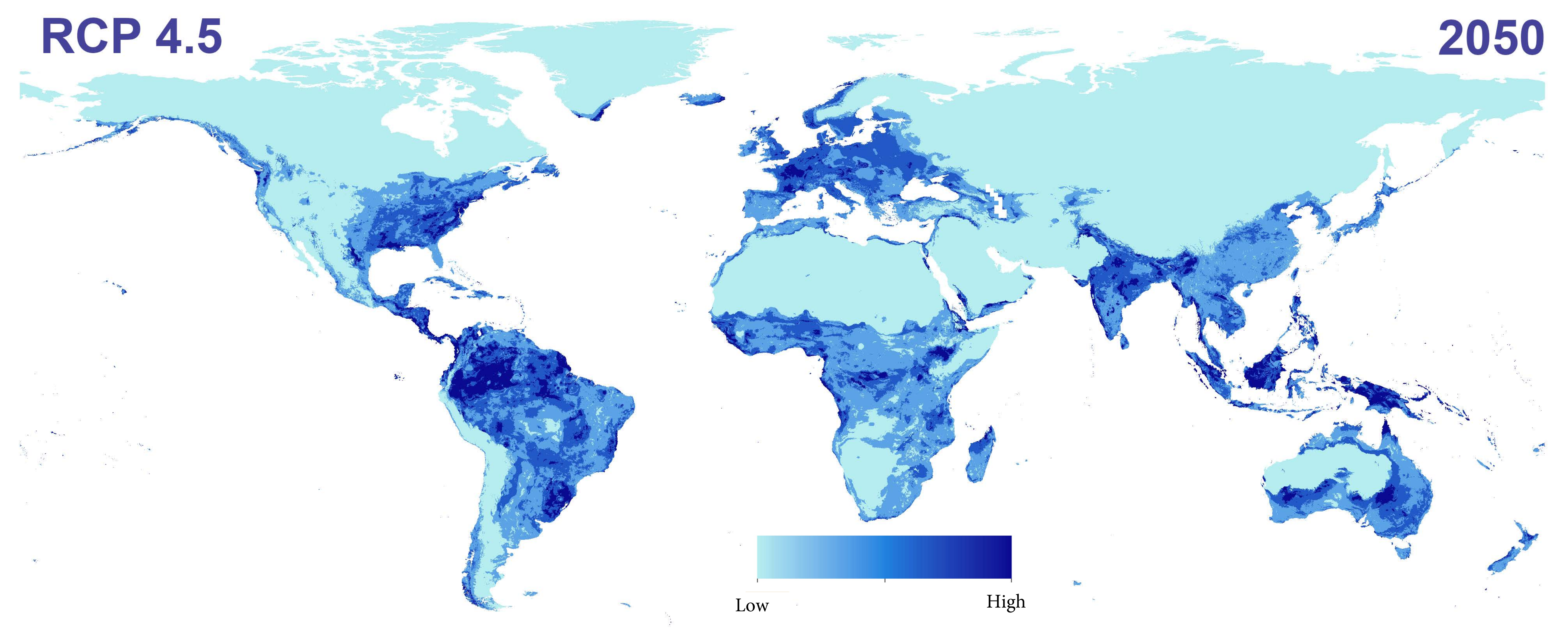
RCP 4.5
2050
Low
High

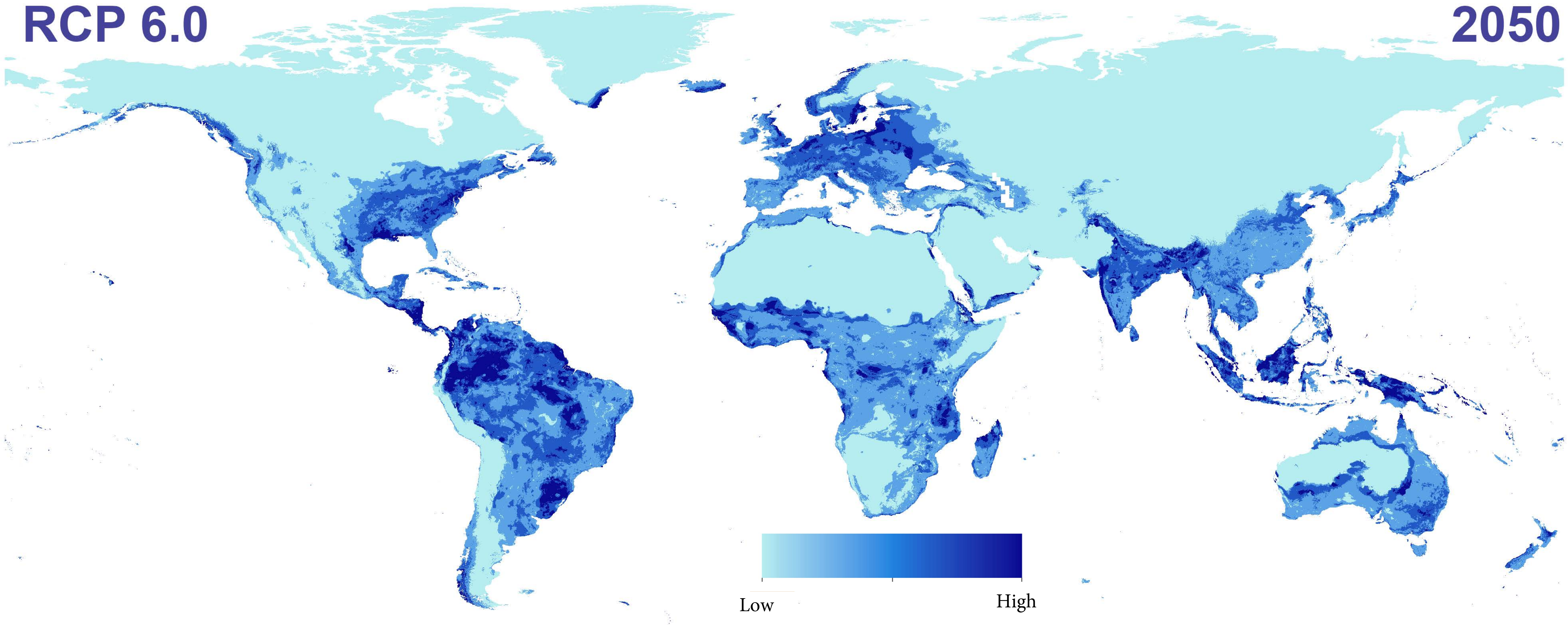
RCP 6.0
2050
Low
High

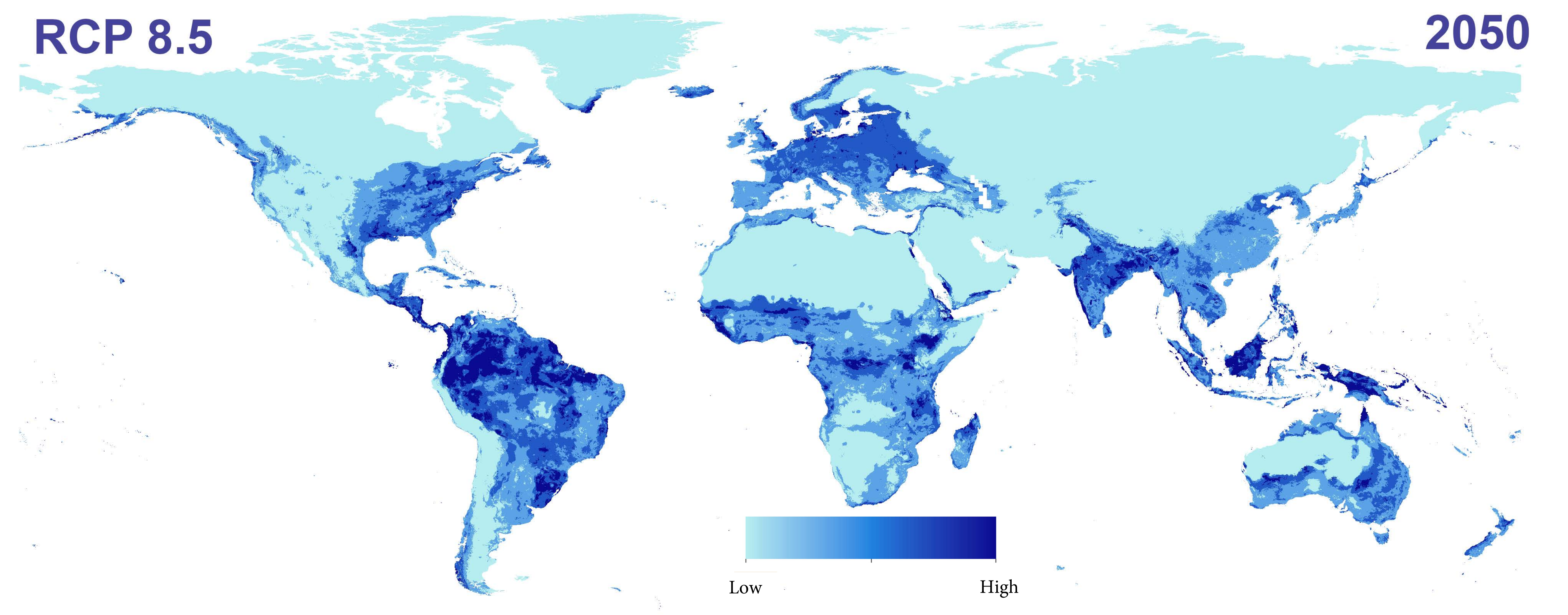
RCP 8.5
2050
Low
High

VI. Visualization of uncertainty estimate associated with future predictions of *Aedes albopictus* based on diverse representative concentration pathways (RCPs) in 2070. Uncertainty index was derived from diverse GCMs in each RCP in the study; uncertainty index was estimated as the range (maximum—minimum) across all combinations of GCMs in each RCP.

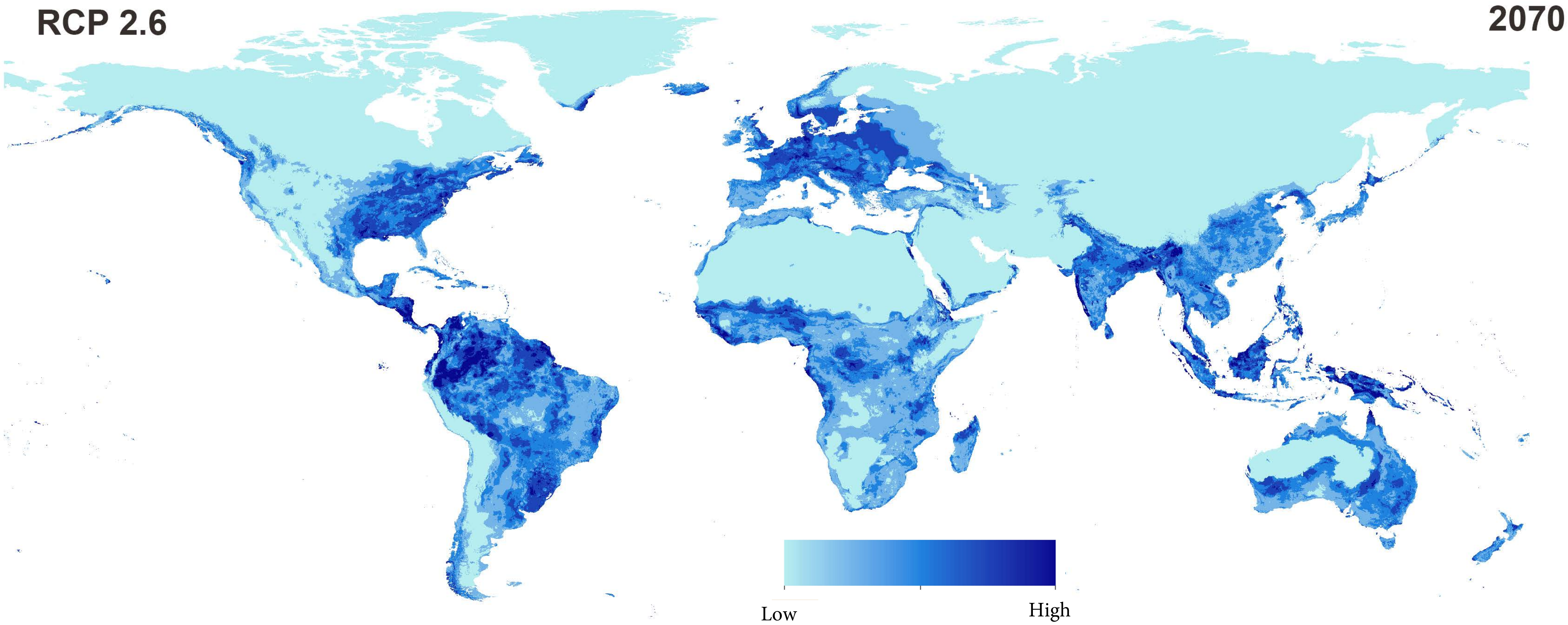

RCP 2.6
2070
Low
High

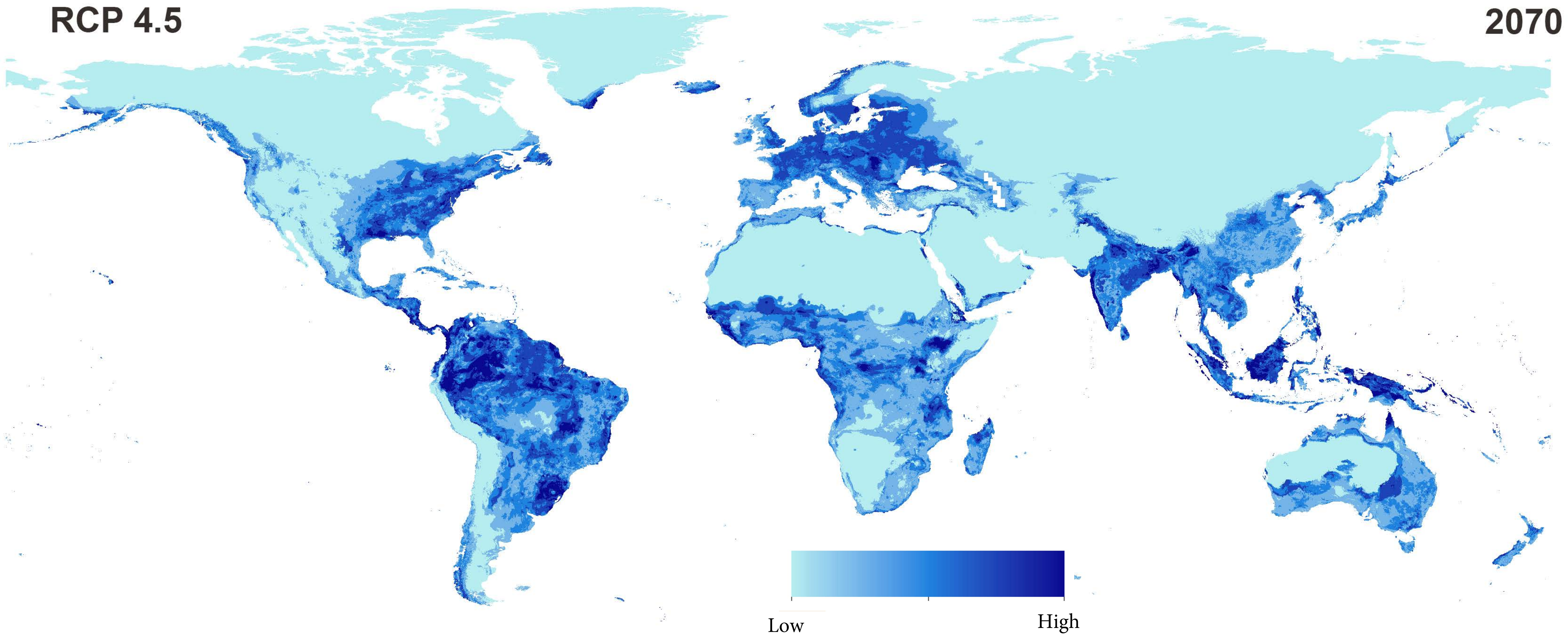
RCP 4.5
2070
Low
High

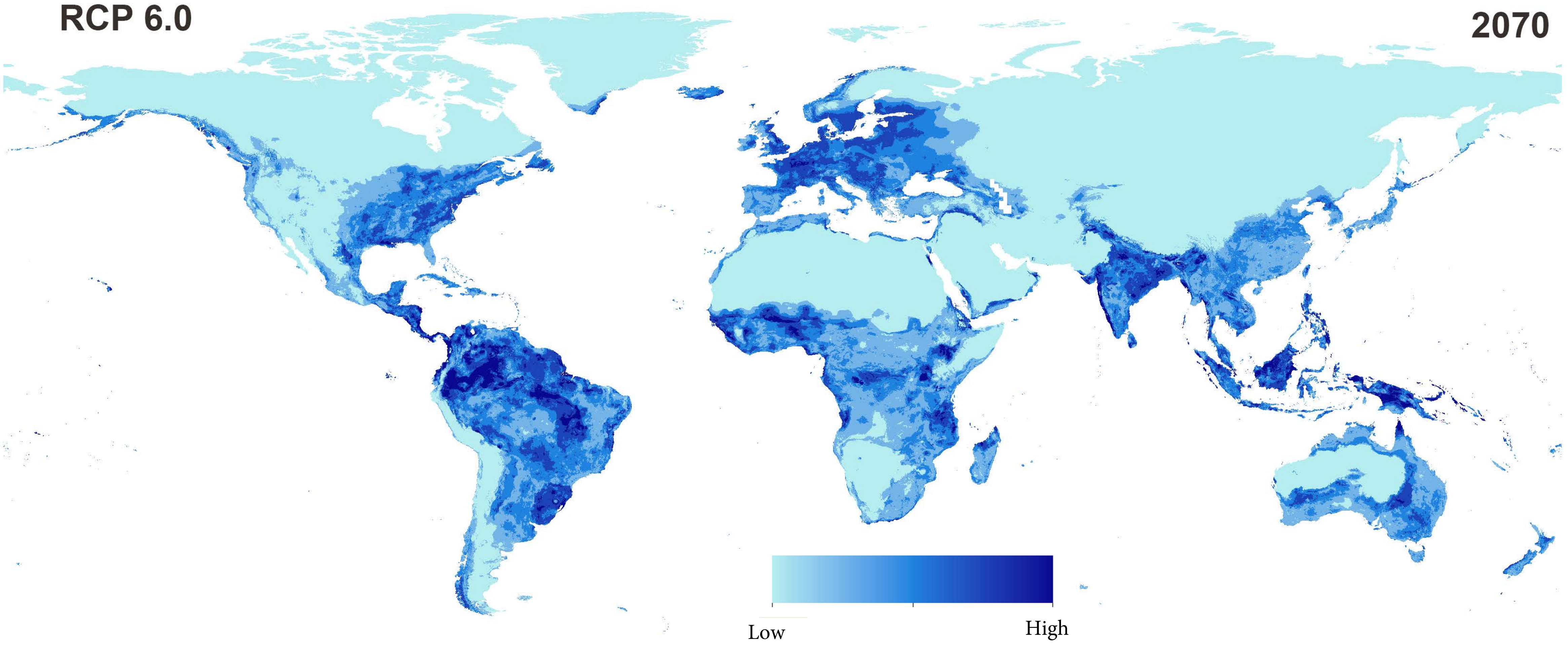

RCP 6.0
2070
Low
High

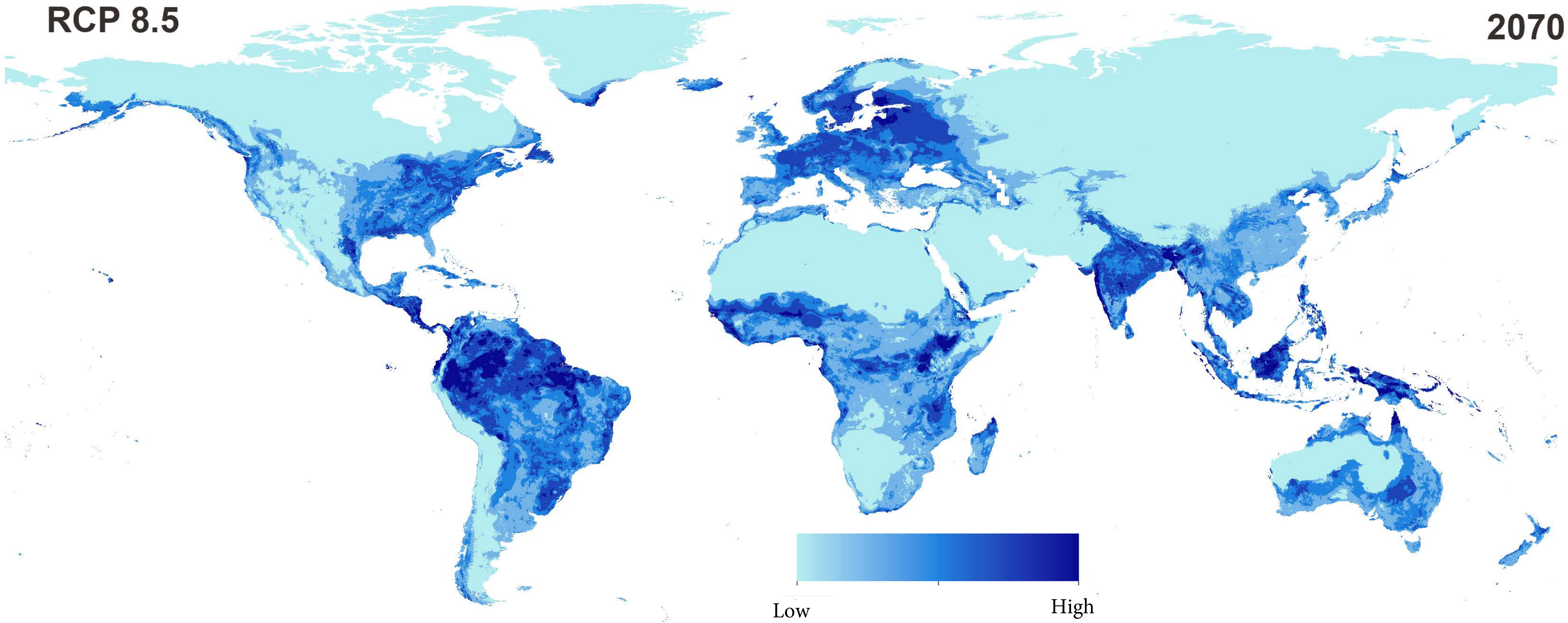
RCP 8.5
2070
Low
High
